# SH3BGRL proteins are thioredoxin fold–containing actin filament pointed end capping proteins (TPECs)

**DOI:** 10.1038/s41598-025-34096-y

**Published:** 2026-01-21

**Authors:** Robin S. Heiringhoff, Daniel Marke, Ute Curth, Johannes N. Greve

**Affiliations:** Institute for Biophysical Chemistry, Hannover Medical School, Fritz-Hartmann-Centre for Medical Research, Hannover, 30625 Germany

**Keywords:** Thioredoxin fold, Actin dynamics, Capping proteins, Actin-binding proteins, Biochemistry, Biophysics, Cell biology, Molecular biology, Structural biology

## Abstract

Cellular actin dynamics are tightly regulated by actin-binding proteins with specialized domains. Here, we identify SH3BGRL proteins as modulators of actin dynamics, characterized by their thioredoxin (Trx) fold and the absence of the canonical enzymatic site. The Trx fold is generally associated with enzymatic activity; however, in this context, it functions non-enzymatically to enhance actin nucleation, inhibit depolymerization and cap the growing pointed end of the actin filament. Our results indicate that all SH3BGRL isoforms weakly promote actin nucleation *in vitro* by stabilizing energetically unstable actin dimers and trimers. Using molecular dynamics simulations and assays that directly probe the pointed end of the actin filament, we show that SH3BGRL proteins efficiently inhibit actin subunit addition at the pointed end by direct association with the terminal actin subunits. However, SH3BGRL proteins are less effective at preventing subunit loss from the pointed end, but they can cooperate with tropomodulin to enhance this activity in an isoform-specific manner, indicating that all isoforms are capable of forming a tripartite complex with actin and tropomodulin. Based on our results, we propose a new and more appropriate name that reflects the function of the SH3BGRL protein family: **t**hioredoxin fold–containing **p**ointed **e**nd **c**apping proteins (TPECs).

**Supplementary Information:**

The online version contains supplementary material available at 10.1038/s41598-025-34096-y.

## Introduction

Members of the SH3-binding glutamic acid rich (SH3BGR) protein family are small proteins, ranging from 10 kDa to 20 kDa in size. They are characterized by a common thioredoxin (Trx) fold, but notably lack a catalytic CXXC or CXXS/T motif canonically associated with Trx fold containing enzymes like thioredoxins, protein disulfide-isomerases, glutaredoxins, glutathione S-transferases, and glutathione peroxidases^1–3^.

**Fig. 1:**
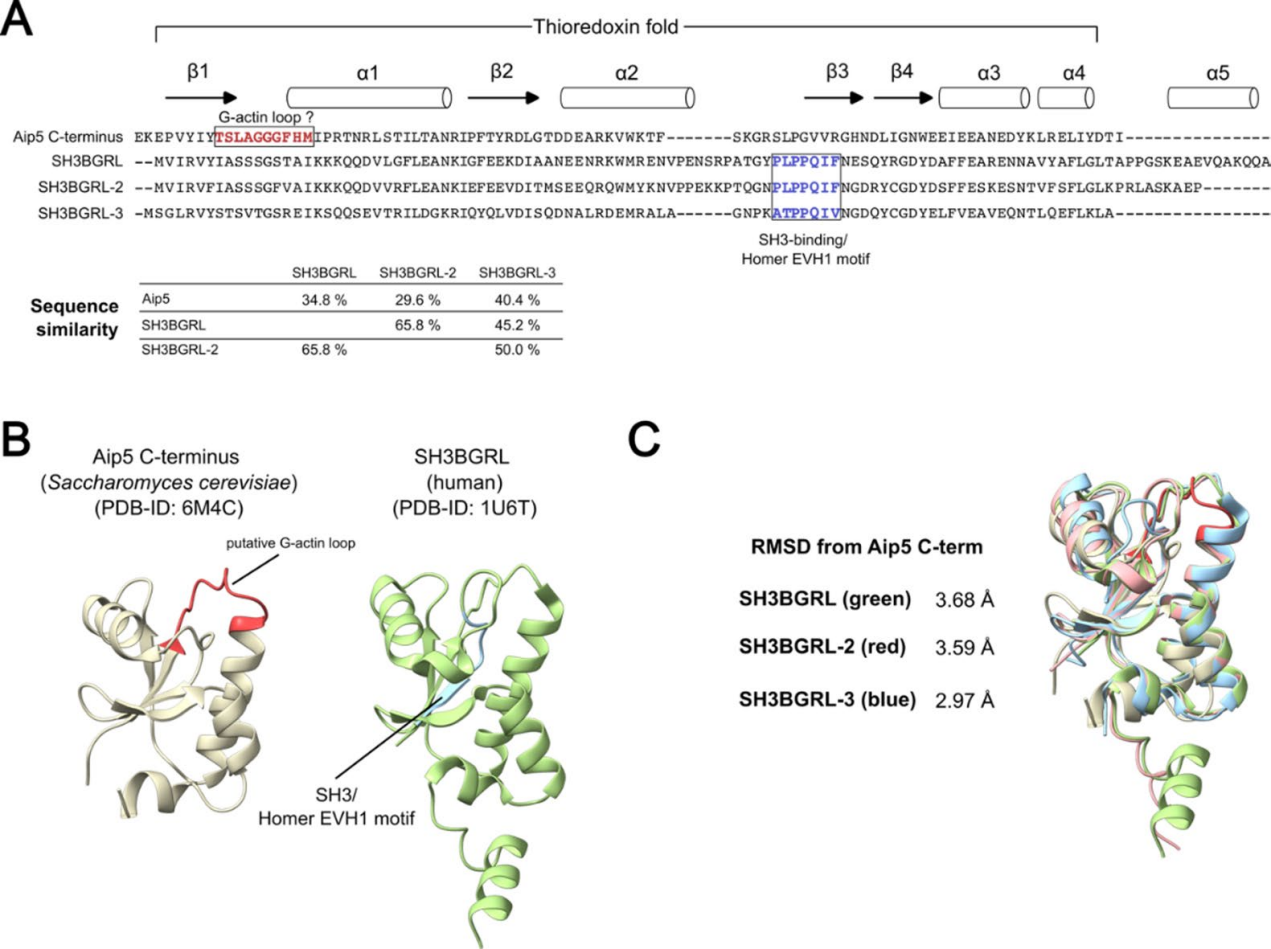
Sequence and structure comparison of the human SH3BRGL isoforms and the C-terminal region of Aip5 from *Saccharomyces cerevisiae.* **(A)** Sequence alignment of the human SH3BGRL isoforms and the Aip5 C-terminus (generated using Clustal Omega^11^. The putative G-actin loop in Aip5 and the SH3-binding/Homer EVH1 motif in SH3BGRL isoforms are highlighted in the sequence. Sequence similarities were determined using the Sequence Manipulation Suite^12^ and are given in the table. **(B)** Published experimental structures of the Aip5 C-terminus from *Saccharomyces cerevisiae* and of human SH3BGRL. The putative G-actin loop in Aip5 and the SH3-binding/Homer EVH1 motif in SH3BGRL are highlighted in the structure. **(C)** Structural alignment of the Aip5 C-terminus with the human SH3BGRL isoforms using the experimental structures of Aip5 C-terminus, SH3BGRL, SH3BGRL-3 and an AlphaFold-generated model of human SH3BGRL-2. The RMSD values were determined using the matchmaker function in ChimeraX^13^.

The eponymous member of the family, the cardiac and skeletal muscle-specific SH3BGR, is further distinguished by a C-terminal extension that is rich in glutamic acid residues^4,5^. The remaining three members of the SH3BGR family, named SH3-binding glutamic acid rich-like/-2/-3 (SH3BGRL/-2/-3), are ubiquitously expressed and lack the C-terminal extension^6–8^. SH3BGR, SH3BGRL and SH3BGRL-2 all feature a single proline-rich sequence (PLPPQIF), which contains the eponymous SH3-binding motif (PXXP) and a Homer EVH1 binding motif (PPXXF). The shortest family member, SH3BGRL-3, contains only the Homer EVH1 binding motif^9,10^. In all cases binding of proteins to these motifs is unlikely, as the motifs are buried inside the tertiary structure^10^ (Fig. 1B).

The absence of the catalytic motif in the otherwise structurally conserved Trx fold raises the question of the functional relevance of the SH3BGR protein family. Recently, a study combining cryo-electron microscopy with mass spectrometry has provided direct visual evidence of the binding of SH3BGRL-2 to actin filaments within the native spectrin–actin complex isolated from porcine erythrocytes^14^. In this multi-protein complex, SH3BGRL-2 is located at the pointed end of the actin filament alongside the capping protein tropomodulin, where it is thought to act as an additional capping protein to protect and stabilize the pointed end. This study provided the first direct evidence for a potential binding partner of the SH3BGRL protein family and suggests that these proteins may play a physiological role in regulating actin dynamics.

Previous studies on the family member SH3BGR indicated that production of the protein is important for proper sarcomere organization and maintenance^5,15^. Cellular studies on the ubiquitously produced isoforms mainly place the function of the proteins in the context of carcinogenesis, either reporting expression as oncogenic^16–18^ or anti-oncogenic^16,19,20^, depending on isoform and species. Furthermore, studies in zebrafish indicate relevance of SH3BGRL for female fertility^21^ and of SH3BGRL, SH3BGRL-2 and SH3BGRL-3 for organogenesis throughout different stages of development^22^. A study reported direct interaction between SH3BGRL-3 and human myosin-1C by performing co-immunoprecipitation and mass spectrometry^23^. The same study reported that knocking down SH3BGRL-3 reduced the migratory capacity of MDA-MB-231 breast carcinoma cells, suggesting a potential link between SH3BGRL proteins and actin dynamics in non-muscle cells. In addition, direct interaction between SH3BGRL and ribosomal subunits has been reported^18^.

The widespread production of the SH3BGRL isoforms and the evident direct interaction of the SH3BGRL-2 isoform with actin filaments indicate a direct involvement of these proteins in the maintenance and regulation of cytoskeletal actin dynamics, potentially extending beyond erythrocytes to other non-muscle cells. In this study, we utilize an integrated approach combining bioinformatics and *in vitro* biochemical analysis with purified proteins to explore the potential roles of human SH3BGRL isoforms in modulating actin dynamics.

## Results

### SH3BGRL proteins and the C-terminal region of actin-interacting protein 5 (Aip5) from *Saccharomyces cerevisiae* share a common thioredoxin fold

Actin-binding proteins utilize a variety of conserved protein folds to interact with both monomeric (G-) actin and filamentous (F-) actin. Among the most common folds are the actin-depolymerizing factor/cofilin (ADF/cofilin) domain fold, the gelsolin-homology domain fold, and the myosin motor domain fold^24^. The thioredoxin (Trx) fold, however, is not widely recognized as a major actin-binding fold.

We initially searched the BioGRID database to identify binding partners of the SH3BGRL protein family in cell types that do not possess the specialized spectrin–actin complexes found in erythrocytes^25^. Deposited interactomes derived from HCT116 and HKe-3 colon cancer cell lines, as well as HEK293 cells include numerous proteins directly associated with the actin cytoskeleton in the datasets for SH3BGRL and SH3BGRL-2 (Supplementary Table 1)^26–28^.

Next, to identify potential structural homologues implicated in actin dynamics, we conducted a protein structure search in the Protein Data Bank (PDB) using the SH3BGRL-2 sequence as the query in Foldseek^29^. Among many thioredoxin and glutaredoxin protein structures, we identified the ordered C-terminal region of actin-interacting protein 5 (Aip5) from *Saccharomyces cerevisiae* (PDB ID: 6M4C) as a structural homologue to SH3BGRL-2, with a template modeling score of 0.77, an RMSD of 2.63 Å, and 21.2% sequence identity (Fig. 1B).

A structural alignment of the available crystal structure of the Aip5 C-terminus with the crystal structures of human SH3BGRL (PDB ID: 1U6T), human SH3BGRL-3 (PDB ID: 1T1V), and an AlphaFold-generated model of human SH3BGRL-2 revealed significant structural homology between the yeast protein fragment and the three human proteins (Fig. 1C). All four structures share the characteristic Trx fold architecture, consisting of a central four-stranded β-sheet flanked by four α-helices, with isoform-specific structural differences confined to their respective C-termini.

### SH3BGRL proteins are monomeric in solution

**Fig. 2:**
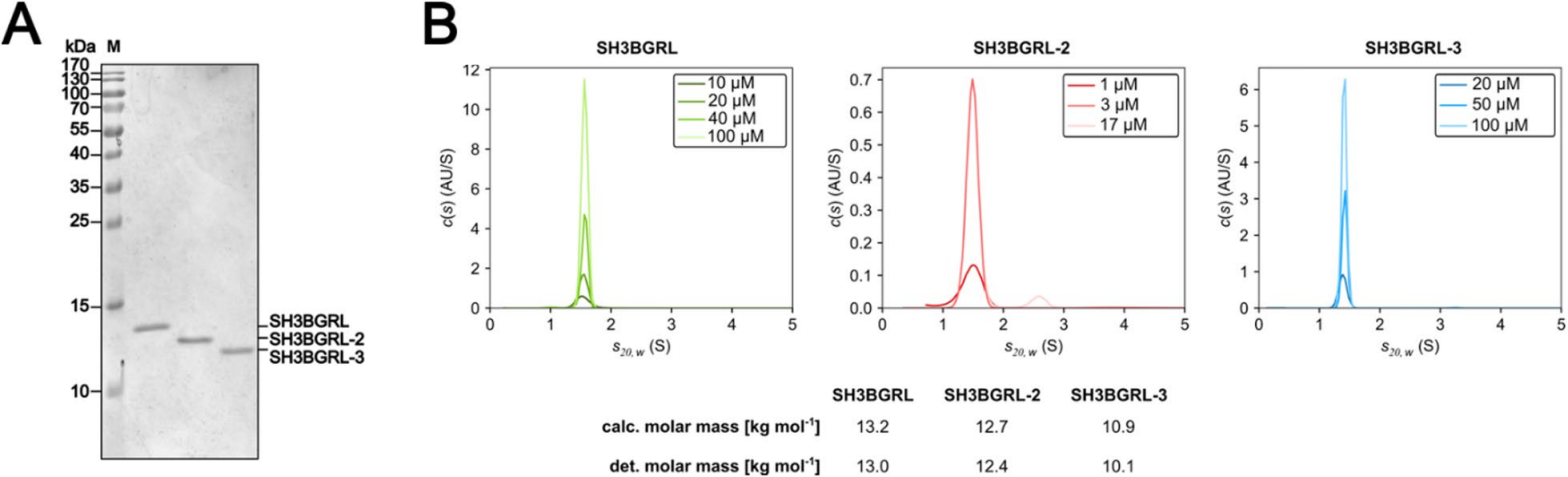
Analysis of the oligomerization state of human SH3BGRL proteins in solution. **(A)** SDS-PAGE of the purified recombinant human SH3BGRL isoforms used for biochemical studies. **(B)** Sedimentation velocity analyses of SH3BGRL, SH3BGRL-2 and SH3BGRL-3 at the indicated protein concentrations. The proteins sediment independently of the protein concentration as single species at s_20,w_=1.55 S (SH3BGRL), s_20,w_=1.49 S (SH3BGRL-2) and s_20,w_=1.41 S (SH3BGRL-3). The experimentally determined molar mass (det.) and the molar mass calculated from the amino acid composition (calc.) are given in the table. Note that the SH3BGRL-2 preparation contains a minor nucleic acid impurity that sediments at about 2.6 S. Because the sedimentation profiles at 1 µM and 3 µM were recorded at a wavelength of 230 nm and the profile at 17 µM was recorded at 280 nm, the minor nucleic acid impurity is only visible in the c(s) distribution at 17 µM.

A precise understanding of the oligomerization state of the SH3BGRL proteins in solution is of importance for our understanding of the SH3BGRL-actin interaction, however, prior work gave inconsistent results^9,10^. We therefore tested the hydrodynamic properties of the purified recombinant human SH3BGRL isoforms (Fig. 2A) by sedimentation velocity experiments using analytical ultracentrifugation (Fig. 2B). Across all tested protein concentrations, SH3BGRL, SH3BGRL-2 and SH3BGRL-3 each sedimented as single species, with sedimentation coefficients s_20,w_ of 1.55 S, 1.49 S and 1.41 S, respectively. Based on the sedimentation coefficients and the diffusional broadening of the sedimenting boundaries, molar masses of 13.0 kg mol^− 1^, 12.4 kg mol^− 1^ and 10.1 kg mol^− 1^, respectively, were determined using the continuous c(s) distribution model implemented in the program SEDFIT^30^. These results agree very well with the masses calculated from the amino acid compositions and clearly support a monomeric state of all three isoforms under the chosen experimental conditions. From the molar masses and the sedimentation coefficients, frictional ratios f/f_0_ of 1.32, 1.30 and 1.27 can be calculated for SH3BGRL, SH3BGRL-2 and SH3BGRL-3, respectively, showing that all three isoforms deviate only moderately from the shape of a sphere.

### SH3BGRL proteins do not interact with G-actin

In the spectrin–actin complex, SH3BGRL-2 inserts between the ultimate and penultimate actin protomers, forming contacts with both, which indicates that efficient actin binding requires two actin molecules^14^ (Suppl. Figure 1). A previous study showed that the structurally related C-terminal region of Aip5 interacts with G-actin through a putative G-actin–binding loop (Fig. 1A, B)^31^. Based on this, we sought to determine whether monomeric human SH3BGRL isoforms can bind monomeric actin with appreciable affinity to form a heterodimeric complex.

We initially used AlphaFold3 and AlphaFold-Multimer to generate models of the potential SH3BGRL/-2/-3 – G-actin complex^32^. However, we observed significant discrepancies between the different AlphaFold versions, as well as a bias toward the arrangement of the penultimate actin protomer and SH3BGRL-2 seen in the available cryo-EM structure of the SH3BGRL-2–decorated actin filament, even when no templates were used. (Supplementary Fig. 2).

Motivated by the discrepancy between the results obtained from different AlphaFold versions, we set out to investigate the potential SH3BGRL/-2/-3 – G-actin complex *in vitro*. In our *in vitro* experiments, we primarily focused on SH3BGRL and SH3BGRL-3, as these proteins could be purified at high yield and concentrated to high concentrations, whereas purifications of SH3BGRL-2 consistently yielded lower amounts.

**Fig. 3:**
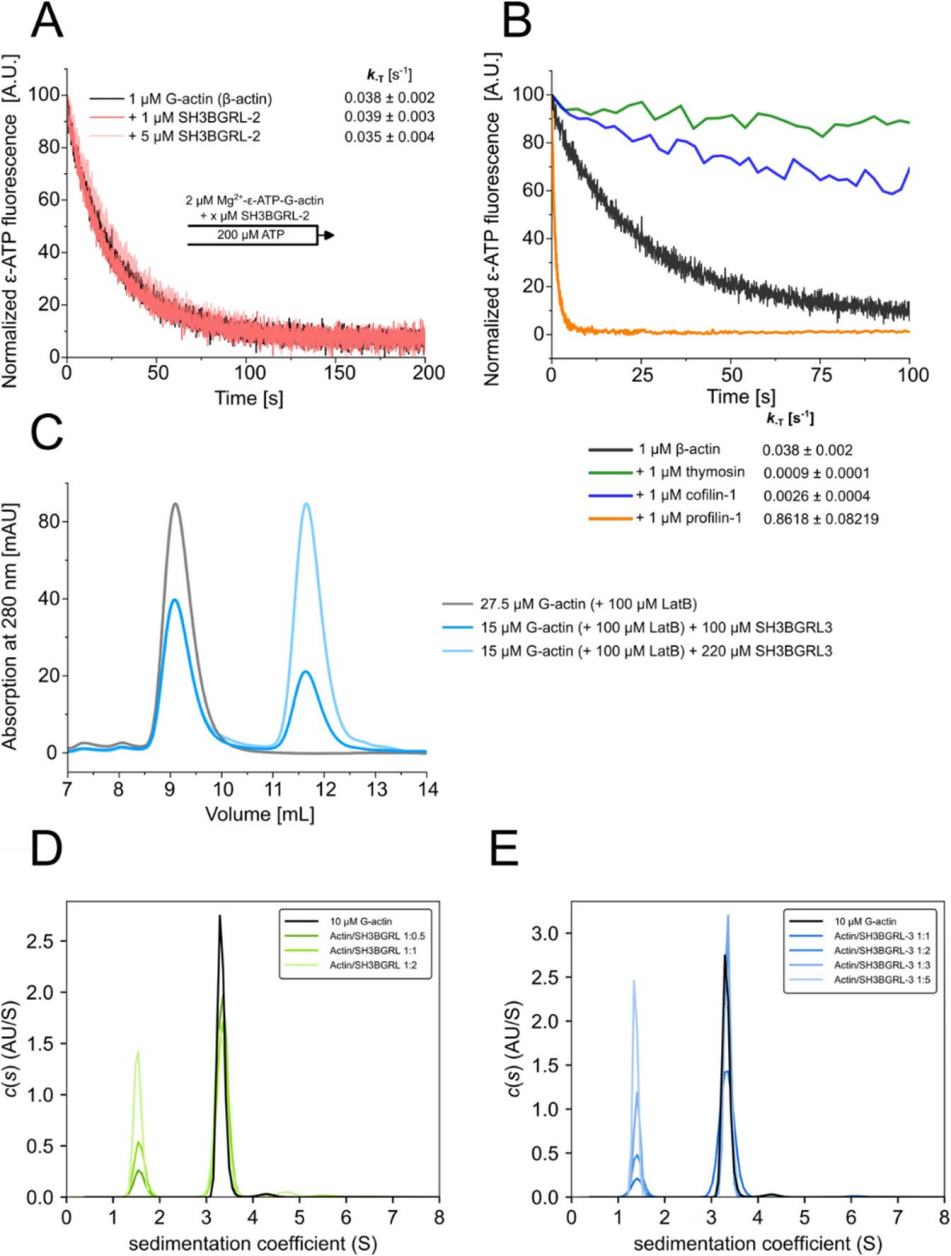
*In vitro* analysis of the potential SH3BGRL–G-actin complex. **(A)** The rate constant of nucleotide dissociation (*k*_− T_) from G-actin was determined for monomeric β-actin in the absence and presence of SH3BGRL-2 in stopped-flow measurements using fluorescently labeled ATP (ε-ATP) in G-buffer. Shown are representative experimental traces. The indicated values for *k*_− T_ are the mean ± SD of 11–12 individual mixing experiments and were determined by fitting mono-exponential functions to the experimental traces. **(B)** Nucleotide dissociation experiments performed with monomeric β-actin and a range of G-actin binding proteins. Experiments were performed and analysed as described in (A). **(C)** Analytical size exclusion chromatography experiments performed with LatB-stabilized G-actin in the absence and in the presence of a high molar excess of SH3BGRL-3 on a S75 10/300 size exclusion chromatography column (GE Healthcare, Chicago, USA) in G-buffer. No shift in the position of the G-actin peak was observed, indicating the absence of a stable SH3BGRL-3–G-actin complex. **(D**,** E)** Sedimentation velocity analysis performed with 10 µM LatB-stabilized G-actin in the absence and presence of increasing concentrations of SH3BGRL (D) or SH3BGRL-3 (E) under buffer conditions stimulating actin assembly (9 mM Tris pH 7.8, 50 mM KCl, 5 mM MgCl_2_, 0.18 mM CaCl_2_, 45 µM ATP, 0.18 mM TCEP). The sedimentation coefficient of the faster sedimenting LatB-stabilized G-actin (3.3 S) did not increase in the presence of the SH3BGRL isoforms, clearly indicating that both proteins do not interact with G-actin under these conditions.

G-actin binding proteins often modulate the exchange of the actin-bound nucleotide with the surrounding environment by either increasing (profilins) or decreasing (thymosin-β4, cofilin) the nucleotide dissociation from G-actin. This exchange can be tracked by using the fluorescent ATP-analogue ɛ-ATP^33^ (Fig. 3B). We performed these experiments in the presence and absence of SH3BGRL-2 (Fig. 3A) to infer possible binding of the isoform to G-actin. However, SH3BGRL-2 had no detectable effect on the dissociation rate constant (*k*_₋T_) of ɛ-ATP from the actin monomer at the low concentrations tested. Next, we performed analytical size-exclusion chromatography experiments using G-actin and a high molar excess of SH3BGRL-3 in the presence of latrunculin B (LatB), a marine toxin inhibiting actin polymerization, in G-buffer (Fig. 3C). We did not observe a shift in the elution volume of G-actin in the presence of SH3BGRL-3, indicating the absence of a stable complex under these non-equilibrium conditions, possibly due to very low affinity.

To rule out the possibility of a weakly interacting complex undetectable by the methods used so far, we performed sedimentation velocity experiments. Unlike size-exclusion chromatography, this technique does not separate the proteins; instead, the faster migrating interaction partner always sediments in a constant bath of the slower one, making the method considerably more sensitive for detecting weakly interacting proteins^34^. In the analysis we used a constant concentration of 10 µM LatB-treated G-actin and increasing concentrations of SH3BGRL or SH3BGRL-3 under buffer conditions commonly used in biochemical studies of actin filament nucleation and elongation. The sedimentation coefficient of actin (3.3 S) did not change in the presence of SH3BGRL or SH3BGRL-3, indicating that SH3BGRL proteins and G-actin do not interact under these conditions (Fig. 3D, E). We conclude that SH3BGRL proteins and monomeric actin do not exhibit appreciable affinity.

### The Trx fold of SH3BGRL isoforms forms a distinctive interface with the F-actin pointed end, with the C-terminus acting as a discriminator between SH3BGRL isoforms

**Fig. 4:**
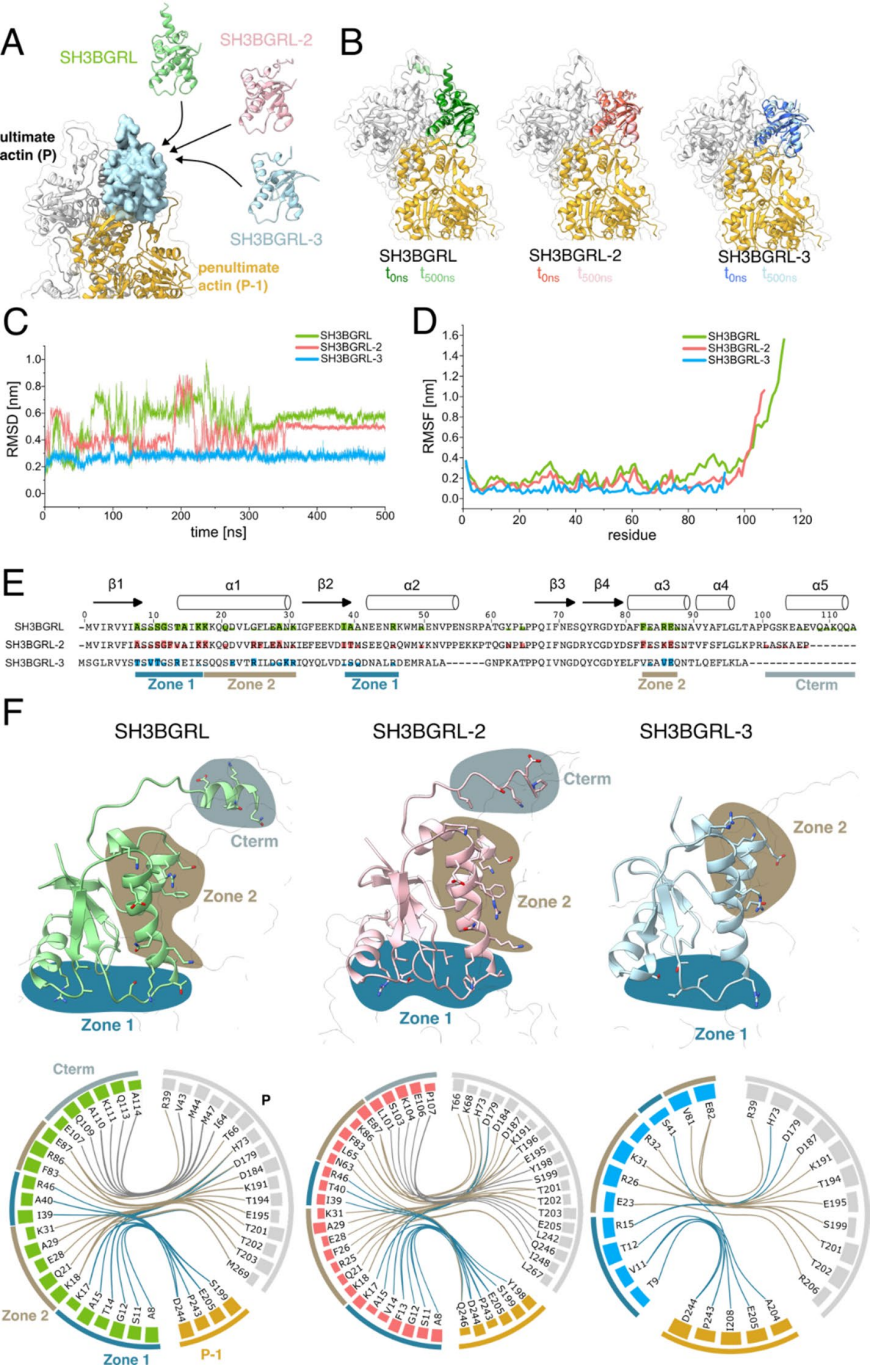
Analysis of the contact interface of SH3BGRL isoforms with the F-actin pointed end using all-atom molecular dynamics simulations. **(A)** The structures of human SH3BGRL isoforms (SH3BGRL. green; SH3BGRL-2, red; SH3BGRL-3, blue) were predicted using AlphaFold and modeled into the position of SH3BGRL-2 at the F-actin pointed end (grey, gold) in the experimental structure of the spectrin–actin complex (PDB-ID: 8IAH). **(B)** Overlay of the first (dark colors) and last frame (light colors) obtained from the molecular dynamics simulations at 0 ns and 500 ns, respectively. **(C)** RMSD values of the SH3BGRL isoforms along the simulation trajectory. **(D)** RMSF values of the SH3BGRL isoforms along the simulation trajectory. **(E)** Sequence alignment of SH3BGRL isoforms, where residues are filled with color according to the fraction of simulation time they spend in contact with actin. Contact zones identified in (F) are labelled. **(F)** Contact analysis of the last 100 ns of the simulation. Contacts are split into zones (Zone 1, Zone 2, Cterm) and are indicated by color. Upper panel: Models of SH3BGRL isoforms at the pointed end after 500 ns simulation, with contact residues shown as stick-style residues. Lower panel: Flareplots showing contacts that exist for at least 50% of the complete simulation time. Residues are shown as nodes and contacts as edges, with the colored bars at the individual residues indicating the relative contact frequency of that specific residue. Edges are colored using the color-scheme from the upper panel.

**Fig. 5:**
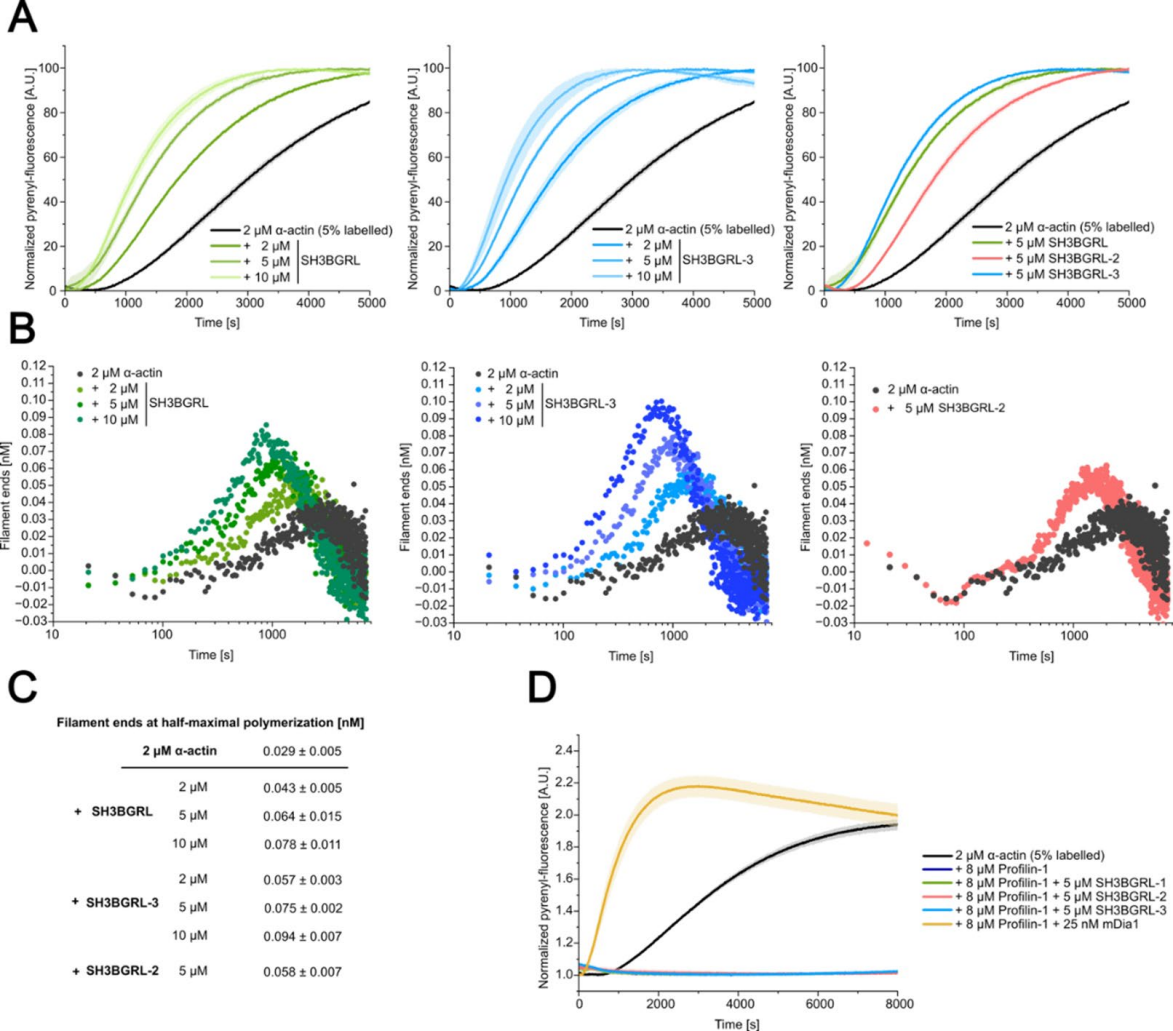
Analysis of the effect of SH3BGRL isoforms on actin polymerization started from G-actin and profilin–G-actin using pyrene-actin based bulk-experiments. **(A)** Pyrene-based polymerization experiments with 2 µM α-actin (5% pyrene-labeled) in the absence and presence of different concentrations of SH3BGRL isoforms. The solid lines/shades represent the mean ± SD of three individual experiments. **(B)** Plots describing the change of filament end concentration over time in the pyrene-actin based bulk-experiments shown in (A). Shown are representative traces. **(C)** Filament end concentrations at half-maximal polymerization. The values correspond to the peak of the traces shown in (B). The concentrations are given as the as the mean ± SD of all performed experiments. *N* = 3 for each condition. **(D)** Pyrene-based polymerization experiments with 2 µM α-actin (5% pyrene-labeled) in the presence of 8 µM profilin-1 and SH3BGRL/−2/−3 or mDia1. The solid lines/shades represent the mean ± SD of three individual experiments.

To comprehensively characterize the interaction between SH3BGRL isoforms and the F-actin pointed end, we performed all-atom molecular dynamics simulations. Initial models were generated by positioning AlphaFold3 models at the position of SH3BGRL-2 in the experimental structure (PDB: 8IAH) (Fig. 4A). Throughout the simulation, the Trx fold of all SH3BGRL isoforms maintained its integrity and showed only minor deviations from the initial model position (Figs. 4B and 5C). The simulation converged to a stable conformation after 350 ns, as indicated by the stable root-mean-square deviation (RMSD) from this point onwards (Fig. 4C). The observed flexibility prior to convergence primarily originated from the C-terminal regions of SH3BGRL and SH3BGRL-2, which are absent in SH3BGRL-3 (Fig. 4D).

To identify key residues at the interaction interface between SH3BGRL isoforms and F-actin, we analyzed which residues made contact with actin throughout the simulation (Supplementary Fig. 3). By combining this analysis with sequence alignment of the three isoforms, we correlated amino acid differences with variations in the binding interface. SH3BGRL, SH3BGRL-2 and SH3BGRL-3 showed similar interaction profiles, with the C-terminal stretch being the most significant difference (Fig. 4E, F; Supplementary Fig. 3).

For a more systematic characterization of the interaction interface, we divided the interaction profiles into three distinct zones (Zone 1, Zone 2, and Cterm) and visualized key interactions (present for > 50% of the last 150 ns simulation time) between SH3BGRL isoforms and the F-actin pointed end (Fig. 4F). The residues in Zone 1 mainly interact with the penultimate protomer (P-1). They involve the N-terminal part from A8 to K17 in SH3BGRL and SH3BGRL-2 and from T9 to R15 in SH3BGRL-3. Three additional residues (I39, A40/T40 and R46) between β-sheet 2 and α-helix 2 also contribute to Zone 1 and only bind to the penultimate protomer (Fig. 4F, Supplementary Fig. 3). In the SH3BGRL-3–F-actin complex, this contact is facilitated by S41. Only residue 15, located at the N-terminal tip of α-helix 1 represents a conserved contact to the ultimate protomer in Zone 1 in all three isoforms.

Zone 2 consists of residues located in α-helix 1 and α-helix 3, which contain predominantly charged residues that interact with the ultimate protomer (P) (Fig. 4E, Supplementary Fig. 3). While SH3BGRL and SH3BGRL-2 show very similar interactions in this zone, SH3BGRL-3 shows fewer interactions. This is mainly due to a slight tilt in α-helix 1 that prevents the formation of contacts with actin at the N-terminal part of this helix (Fig. 4F).

The C-terminal stretch (Cterm) of SH3BGRL and SH3BGRL-2 forms the third interaction motif. Although the conserved Trx folds of both isoforms engage with the pointed end in a similar manner, their C-terminal regions differ notably. The Cterm of SH3BGRL-2 is shorter by seven amino acids compared to that of SH3BGRL. While both isoforms interact with the terminal protomer, they contact distinct residues. The SH3BGRL Cterm interacts with V43, M44, M47, and I63, whereas the SH3BGRL-2 Cterm contacts T194, Y198, S199, E205, L242, Q246, and I248 (Fig. 4F).

### SH3BGRL isoforms stimulate actin polymerization from G-actin but not from profilin–G-actin

Actin polymerization can be described as a two-step process in which a thermodynamically and kinetically unfavorable nucleation step, the formation of an actin-trimer, is followed by a more favorable asymmetric elongation of the trimer into an actin filament^35^.

We hypothesized that SH3BGRL proteins might influence actin polymerization initiated from G-actin by interacting with newly formed oligomers and filaments. To challenge our hypothesis, we performed pyrene-actin based bulk-polymerization assays with pyrene-labeled α-actin and increasing concentrations of human SH3BGRL and SH3BGRL-3 (Fig. 5A). These experiments demonstrated a concentration-dependent stimulatory effect of both isoforms on actin polymerization, displaying the characteristic features of actin nucleators. Specifically, they shortened the initial lag time of the reaction and enhanced the subsequent linear increase in pyrenyl-fluorescence, which is typically correlated with the net polymerization rate of actin filaments, up to 3-fold (Fig. 5A, Supplementary Fig. 4). We repeated the experiments using a specific concentration of SH3BGRL-2, which demonstrated a similar but less pronounced effect on actin assembly. To more precisely quantify the nucleating properties of the SH3BGRL proteins, we calculated the concentration of filament ends over the time-course of the polymerization reaction (Fig. 5B, C; see “Material and methods”). The analysis reveals a concentration-dependent effect of all SH3BGRL isoforms on the concentration of filament ends over the time-course of the experiment, generally resulting in higher filament end concentrations at earlier time-points when compared to experiments without SH3BGRL proteins.

*In vivo*, a significant amount of the available G-actin is bound to members of the profilin protein family. The profilin–G-actin complex constitutes the predominant polymerization-competent actin species in the cytosol, which can be used by actin polymerases like formins to nucleate and elongate filaments^36^. We therefore analysed if the SH3BGRL isoforms can stimulate actin polymerization started from profilin–G-actin by repeating our assays in the presence of an excess of profilin-1 (Fig. 5D). Polymerization traces derived from experiments performed with profilin–G-actin in combination with SH3BGRL isoforms are indistinguishable from those obtained with profilin–G-actin alone, demonstrating that SH3BGRL proteins are unable to counteract profilin-induced inhibition of actin polymerization. Conversely, in control experiments performed with a nanomolar concentration of diaphanous-related formin-1 (mDia1), the formin was able to overcome profilin-induced inhibition of spontaneous actin polymerization (Fig. 5D).

### SH3BGRL proteins stimulate filament nucleation but do not affect barbed end elongation

**Fig. 6:**
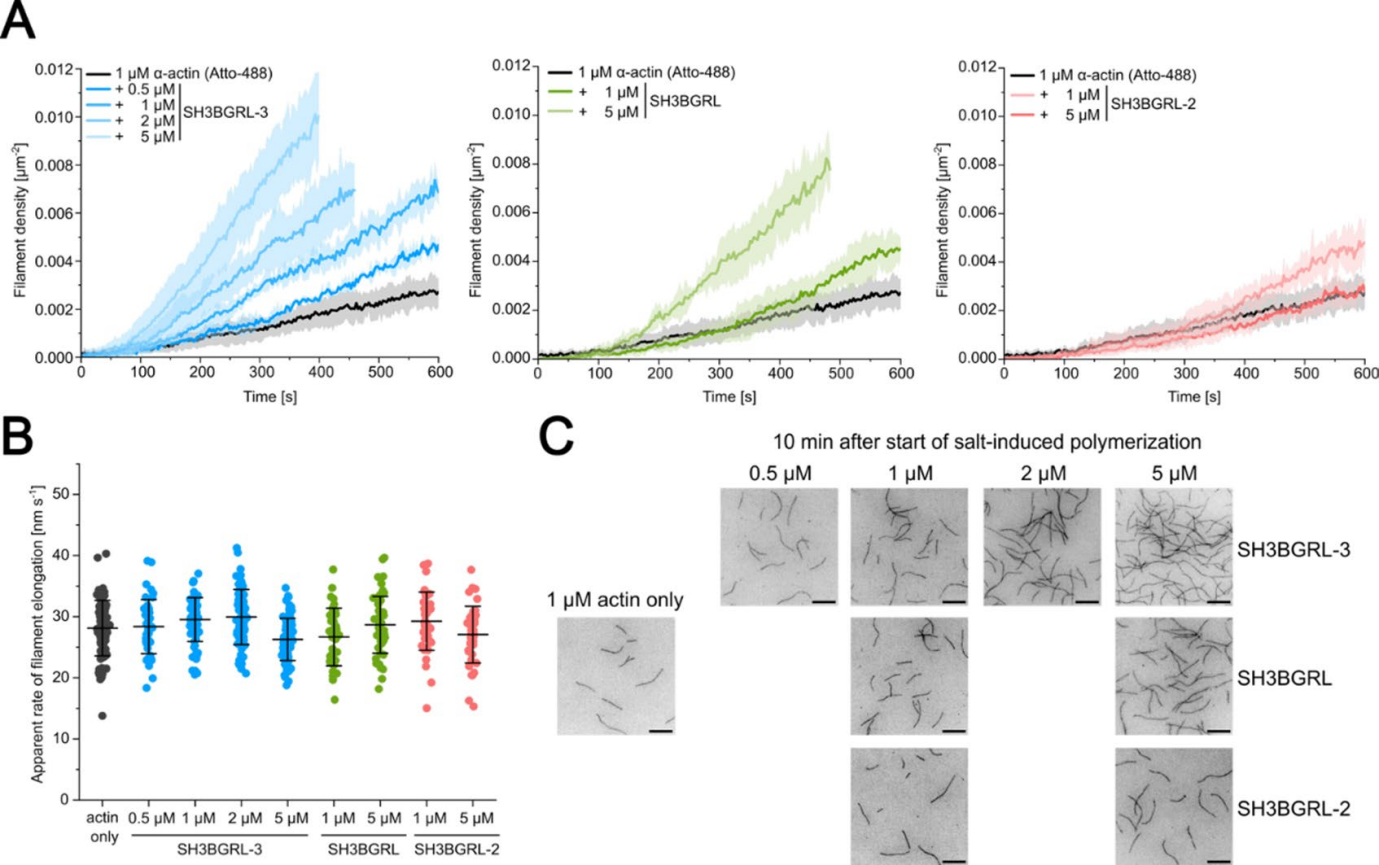
Analysis of the effect of SH3BGRL isoforms on actin nucleation and elongation using *in vitro* TIRF microscopy. **(A)** Polymerization of 1 µM fluorescently labeled α-actin (15% ATTO-488 labeled) was induced by salt-shift in the absence or presence of different concentrations of SH3BGRL isoforms and the progression of the reaction was tracked by TIRF microscopy. The increase in filament density over time was tracked using the *Analyze particles* plugin in ImageJ. The solid lines and shades represent the mean ± SD of at least three individual experiments. Nucleation rates were determined by linear regression analysis of the regions of linear increase. **(B)** The elongation rates of individual filaments were determined by manual tracking of the elongating filaments. Every data point represents an individual filament. Data is shown as the mean ± SD. No significant changes were observed among the various conditions. **(C)** Representative micrographs of TIRFM-based polymerization experiments under the indicated conditions 10 min after induction of polymerization. Scale bar corresponds to 10 μm.

Pyrene-actin based bulk-experiments allow no clear discrimination of actin nucleation and elongation. Therefore, to get a better understanding of the underlying mechanism of SH3BGRL-stimulated actin assembly, we performed single-filament assays with ATTO-488 labeled α-actin using total internal reflection fluorescence microscopy (TIRFM). We analyzed the effect of SH3BGRL-3 on actin polymerization in single-filament assays by titrating a constant concentration of monomeric ATTO-488 labeled α-actin (1 µM) with increasing concentrations of SH3BGRL-3 in G-buffer. Actin polymerization was induced by salt-shift, the reaction mixture flushed into a flow-cell and imaging was initiated. We tracked the growth of individual actin filaments under the different experimental conditions to investigate the effect of the SH3BGRL proteins on filament elongation and repeated the experiments with selected concentrations of SH3BGRL and SH3BGRL-2 (Fig. 6A, C). We observed no significant effect of all three isoforms on the apparent rate of filament elongation (Fig. 6B), which under the experimental conditions is predominantly determined by the elongation of the barbed end. All SH3BGRL proteins significantly increased the filament density over the entire duration of the experiments in a concentration-dependent manner compared to experiments performed with actin only (Fig. 6C). We determined the apparent rate of filament nucleation (*k*_nuc_) by linear regression analysis of the linear regions of the filament density time course (Fig. 6A; Table 1). At the highest concentration used (5 µM), SH3BGRL-3 increased *k*_nuc_ 6.7-fold compared to experiments performed with actin alone. While SH3BGRL showed comparable effects on *k*_nuc_, the stimulating effect of SH3BGRL-2 on actin nucleation was significantly lower, which is in line with the results obtained from the pyrene-actin bulk-experiments. Most of our biochemical studies to this point were performed using α-actin from chicken pectoralis major muscle, as this isoform is easily obtained directly from tissue with high purity and yield and is routinely used in studies of actin dynamics. To assess whether SH3BGRL isoforms can also modulate actin dynamics of other, physiologically more relevant isoforms, we repeated our TIRFM-based experiments using purified recombinant human cytoskeletal β-actin and a selected concentration of SH3BGRL-3 (1 µM). We observed a similar but stronger stimulation of actin nucleation compared to experiments performed with α-actin and the same concentration of SH3BGRL-3 (Supplementary Fig. 5).

**Table 1:** Actin filament elongation and nucleation rates in the absence and presence of SH3BGRL/-2/-3 determined from TIRFM-based experiments.

Parameter	SH3BGRL	SH3BGRL-2	SH3BGRL-3
Apparent rate of filament elongation [nm s^− 1^]	28.1 ± 4.5 (actin only)	28.1 ± 4.5 (actin only)	28.1 ± 4.5 (actin only)
	-	-	28.4 ± 4.4 (0.5 µM)
	26.7 ± 4.7 (1 µM)	29.3 ± 4.8 (1 µM)	29.5 ± 3.6 (1 µM)
	-	-	29.9 ± 4.5 (2 µM)
	28.7 ± 4.6 (5 µM)	27.1 ± 4.6 (5 µM)	26.3 ± 3.5 (5 µM)
Apparent rate of filament nucleation *k*_nuc_ [µm^− 2^ s^− 1^]	5.1 × 10^− 6^ ± 1.1 × 10^− 6^ (actin only)	5.1 × 10^− 6^ ± 1.1 × 10^− 6^ (actin only)	5.1 × 10^− 6^ ± 1.1 × 10^− 6^ (actin only)
	-	-	10.5 × 10^− 6^ ± 0.8 × 10^− 6^ (0.5 µM)
	12.3 × 10^− 6^ ± 1.2 × 10^− 6^ (1 µM)	7.0 × 10^− 6^ ± 1.2 × 10^− 6^ (1 µM)	13.8 × 10^− 6^ ± 2.5 × 10^− 6^ (1 µM)
	-	-	18.4 × 10^− 6^ ± 3.0 × 10^− 6^ (2 µM)
	22.2 × 10^− 6^ ± 3.0 × 10^− 6^ (5 µM)	12.0 × 10^− 6^ ± 1.4 × 10^− 6^ (5 µM)	34.2 × 10^− 6^ ± 6.0 × 10^− 6^ (5 µM)

Taken together, our data from the TIRFM-based experiments and our pyrene-actin based bulk-assays indicate that SH3BGRL proteins stimulate actin assembly solely by increasing the rate of filament nucleation, most likely by stabilizing energetically unstable actin dimers and trimers. In our TIRFM-based experimental setup, the lack of surface-tethered actin filaments, together with the faster growth of the exposed barbed end, limited our ability to analyze elongation at the pointed end in the absence or presence of SH3BGRL proteins. Therefore, we next set out to investigate the effect of SH3BGRL proteins on actin filament pointed end elongation.

### SH3BGRL proteins efficiently inhibit the elongation of the pointed end

**Fig. 7:**
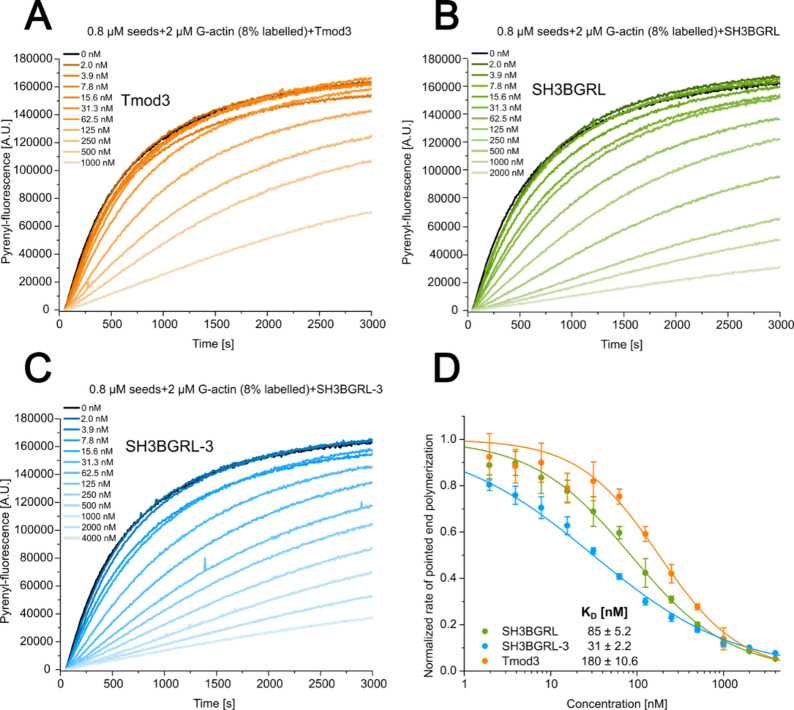
Analysis of the effect of SH3BGRL proteins on pointed end elongation using pyrene-actin based pointed end elongation assays. **(A)** Pyrene-based pointed end polymerization experiments started from 0.8 µM CP-capped F-actin seeds with 2 µM G-actin (α-actin, 8% pyrene-labeled) in the absence and presence of increasing concentrations of Tmod3. Shown are representative traces of at least 3 individual experiments for each condition **(B**,** C)** Pointed end polymerization experiments performed in the presence of increasing concentrations of SH3BGRL (B) and SH3BGRL-3 (C), as described for (A). **(D)** The apparent affinities (*K*_D_) of SH3BGRL/−3 and Tmod3 for the pointed end under the experimental conditions were estimated by plotting the normalized pointed end polymerization rates against the used protein concentration and fitting a hyperbolic function to the complete dataset.

We performed pyrene-actin based bulk-experiments starting from actin filament seeds capped at the barbed ends with human capping protein (CP) to investigate the effect of the SH3BGRL proteins on pointed end elongation. We initially tested our experimental setup by titrating human tropomodulin-3 (Tmod3), a well-characterized pointed end capping protein^37,38^ (Fig. 7A). We observed a strong inhibition of pointed end elongation by Tmod3 across the tested concentration range. From the data, we calculated an apparent dissociation constant (*K*_D_) of 180 ± 10.6 nM for pointed end binding under our experimental conditions (Fig. 7D). This values is in good agreement with previously determined affinities for tropomodulin using the same pointed end elongation assay^39^. Using the same assay, we next examined the effects of the SH3BGRL and SH3BGRL-3 isoforms on pointed end elongation. We titrated both isoforms over a wide concentration range and observed strong concentration-dependent inhibition of pointed end elongation for both isoforms (Fig. 7B, C). We determined apparent *K*_D_ values of 85 ± 5.2 nM for SH3BGRL and 31.0 ± 2.2 nM for SH3BGRL-3 (Fig. 7D). Thus, both isoforms efficiently inhibit pointed end elongation at nanomolar concentrations under the assay’s polymerization-promoting conditions, consistent with a model where their binding sterically blocks further monomer addition at the pointed end.

### SH3BGRL proteins weakly inhibit actin pointed end depolymerization and display isoform-specific cooperativity with tropomodulin

**Fig. 8:**
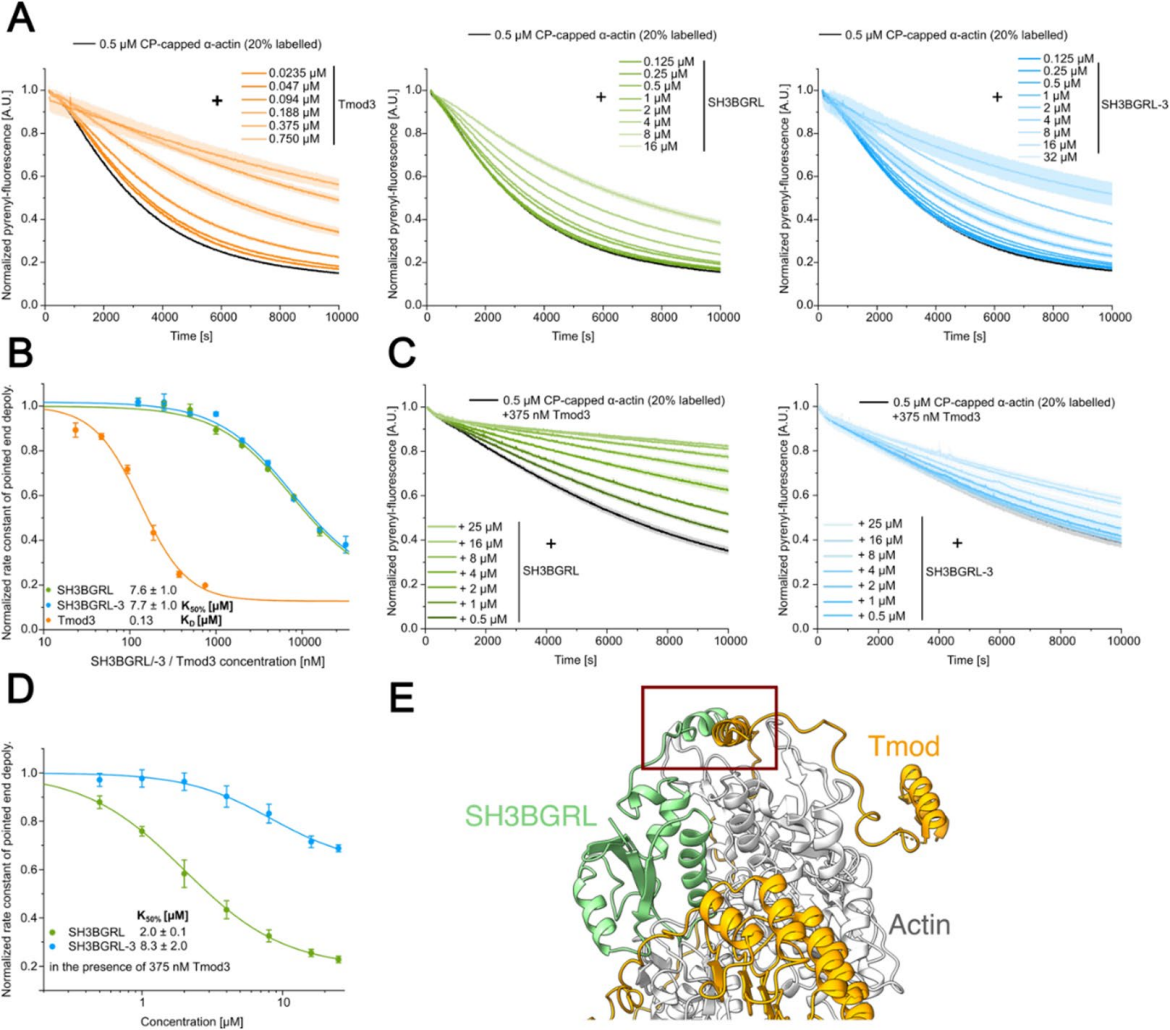
Analysis of the effect of SH3BGRL proteins on pointed end depolymerization in the absence and presence of tropomodulin. **(A)** Pyrene-actin based dilution-induced depolymerization experiments using filaments capped at the barbed end by human capping protein (CP) were used to specifically monitor the effect of Tmod3 and SH3BGRL isoforms on pointed end depolymerization. Data are shown as the mean ± SD. *N* = 3 for each condition. **(B)** The apparent *K*_D_ to the pointed end (Tmod3) or the concentration required to achieve half-maximal inhibition of depolymerization (*K*_50%_, SH3BGRL/−3) under the experimental conditions were estimated by plotting the normalized pointed end depolymerization rate constants against the used protein concentration and fitting a hyperbolic function to the complete dataset. **(C)** Traces of dilution-induced depolymerization experiments performed with pyrene-labelled CP-capped actin filaments and mixtures of Tmod3 (375 nM) and increasing concentrations of SH3BGRL or SH3BGRL-3. **(D)** *K*_50%_ values of SH3BGRL/−3 in the presence of Tmod3, determined as described for (B). **(E)** Composition of the last frame of the SH3BGRL–F-actin MD simulation and Tmod1 of the published spectrin–actin complex structure. All other actin-binding proteins besides Tmod1 were removed from the published structure to ease visualization. The possible contact site between the C-terminal region of SH3BGRL and Tmod is indicated. Please note that Tmod1 has been identified in the published structure, but we used the ubiquitously produced isoform Tmod3 for our assays.

As our experiments indicate efficient inhibition of pointed end elongation by SH3BGRL and SH3BGRL-3, we next sought to determine whether SH3BGRL proteins can protect the pointed end from depolymerization. To this end, we performed depolymerization studies with F-actin capped at the barbed end by CP. We polymerized pyrene-labelled actin in the presence of CP and followed the depolymerization by diluting the sample below the critical concentration of the pointed end (~ 0.6 µM). Again, we initially tested our experimental setup by titrating Tmod3. We observed a pronounced, Tmod3 concentration-dependent reduction in the apparent rate constant of pointed end depolymerization, indicating that Tmod3 associates with the pointed end and protects it from depolymerization (Fig. 8A), which allows us to estimate the *K*_D_ of the Tmod3–pointed end complex under these experimental conditions to be 0.13 ± 0.02 µM (Fig. 8B). This value is in good agreement with previous *K*_D_ values obtained using similar methods^37^, as well as with the *K*_D_ value determined earlier in this study from inhibition of pointed end elongation. Next, we examined the ability of the SH3BGRL proteins to protect the pointed end from depolymerization by titrating SH3BGRL and SH3BGRL-3. Both proteins showed a similar but significantly weaker effect on the pointed end depolymerization, when compared to Tmod3 (Fig. 8A). We estimated the concentrations required to achieve half-maximal inhibition of depolymerization (*K*_50%_) to be 7.6 ± 1.0 µM (SH3BGRL) and 7.7 ± 1.0 µM (SH3BGRL-3), respectively (Fig. 8B). We chose to use the term *K*_50%_ instead of *K*_D_ to avoid confusion with the apparent *K*_D_ value obtained from pointed end elongation assays, which we assume is closer to the true *K*_D_.

To assess whether SH3BGRL proteins and Tmod3 can simultaneously occupy the pointed end, as previously shown for SH3BGRL-2^14^, we performed experiments using a constant concentration of Tmod3 (0.375 µM) and increasing concentrations of either SH3BGRL or SH3BGRL-3 (Fig. 8C). For SH3BGRL, we observed a stronger inhibition of pointed end depolymerization in the presence of Tmod3, resulting in a lower *K*_50%_ value for SH3BGRL, consistent with cooperative behavior. For the shorter isoform SH3BGRL-3, titration in the presence of Tmod3 also led to enhanced inhibition of pointed-end depolymerization; however, in this case, no change in the *K*_50%_ of SH3BGRL-3 was detected (Fig. 8D). Overall, both SH3BGRL and SH3BGRL-3 inhibited pointed end depolymerization more efficiently when combined with Tmod3 than when tested individually, indicating that SH3BGRL proteins and Tmod do not displace each other at the pointed end.

An overlay of the last frame of the SH3BGRL–F-actin MD simulation and the published spectrin–actin structure, which also contains tropomodulin, supports this notion. Although the C-terminal part of SH3BGRL is in close proximity to tropomodulin, no major steric hindrances are expected, also due to the flexibility of this region observed in the MD simulations (Fig. 8D).

## Discussion

In this study, we explored the interactions between the universally produced SH3BGRL isoforms (SH3BGRL, SH3BGRL-2, SH3BGRL-3) and actin to unveil a potential role for this protein family in modulating actin dynamics. Through analytical ultracentrifugation, we have now demonstrated that all members of the SH3BGRL family exist in the micromolar concentration range as monomers in solution. This finding clarifies previous crystallography-based assumptions that suggested oligomerization for SH3BGRL-3^9^. Our findings are consistent with the published monomeric structure of SH3BGRL-2 at the pointed end of actin filaments^14^, reinforcing the hypothesis that all SH3BGRL isoforms interact with actin through a similar mechanism.

Through a combination of direct and indirect experimental approaches, we found that SH3BGRL proteins show no appreciable affinity for monomeric actin. This is consistent with their structural arrangement at the pointed end of the actin filament, where SH3BGRL proteins contact two actin protomers simultaneously. Although we employed high concentrations of SH3BGRL proteins in selected assays assessing G-actin binding, any potential interaction appears too weak to be detected under our experimental conditions. In vivo, monomeric actin is predominantly bound by profilin or thymosin-β4, which interact with monomeric actin with affinities in the high-nanomolar to low-micromolar range^40^. Therefore, efficient binding of SH3BGRL proteins to monomeric actin in cells is unlikely.

Additionally, we observe nucleating properties in both bulk-polymerization assays and single-filament experiments, but no detectable effect on barbed end elongation. Based on these observations, we propose that SH3BGRL proteins facilitate actin nucleation by stabilizing thermodynamically and kinetically unfavorable actin dimer and trimer states^35^ in a structural arrangement reminiscent of the arrangement of SH3BGRL proteins at the pointed end of the filament, thereby allowing normal barbed end elongation from SH3BGRL-nucleated filaments. Unlike well-characterized actin nucleators, SH3BGRL proteins lack the Wiskott-Aldrich homology 2 (WH2) domain, a ~ 17-amino acid actin-binding motif often present in potent actin nucleators^41^. WH2 domains, often present in tandem repeats, are crucial for actin monomer recruitment and nucleation. Despite the prevalence of WH2 domains among efficient actin nucleators, selected proteins, such as tropomodulin isoforms, can also nucleate actin in their absence, suggesting alternative mechanisms of nucleation^42^. The same study showed that the nucleating properties of selected tropomodulin isoforms can be attributed, at least in part, to their G-actin binding. While tropomodulin and SH3BGRL proteins do not share G-actin binding properties, they have in common that they are weak actin nucleators *in vitro* compared to potent WH2-containing nucleators, which exhibit highly efficient nucleation in the low nanomolar concentration range. Furthermore, we show that SH3BGRL proteins do not nucleate actin when using profilin–actin as a substrate, although this conclusion is based on testing a single SH3BGRL concentration (5 µM). Taken together, these findings suggest that SH3BGRL proteins, if functioning alone, would need to reach very high local concentrations to drive appreciable actin nucleation in cells. Because the cellular and especially subcellular concentrations of SH3BGRL proteins are still unknown, we cannot currently assign a definitive actin-nucleating role to this protein family.

Using molecular dynamics simulations followed by comprehensive contact analysis, we characterized the interaction between the Trx fold and the pointed end of the actin filament across all three isoforms. Despite variations in their primary amino acid sequences, the three SH3BGRL isoforms share similar, though not identical, contact interfaces with the pointed end (Supplementary Fig. 6, Fig. 4E). The variations at the C-terminus represent the most significant differences among the isoforms, yet their influence on pointed end binding remains unclear. When comparing the nucleating effect on actin assembly of the three isoforms, SH3BGRL-2 shows the lowest potency. This seems to contrast with the interaction interface analysis, which shows a very similar interaction interface of SH3BGRL and SH3BGRL-2 with the pointed end. A possible explanation for this discrepancy could be the role of the C-terminal stretch, which is different in the two isoforms. A greater flexibility of this stretch in the SH3BGRL-2 isoform may hinder initial actin binding and thereby reduce the potency of actin nucleation.

It has been shown that the two terminal actin subunits at the pointed end exhibit a twisted conformation between subdomains 1 and 2 and subdomains 3 and 4, resembling monomeric rather than filamentous actin^43,44^. Dihedral angles for P and P-1 in free pointed ends (PDBs: 8F8S, 9FJO) are approximately − 17°. In Tmod1-capped structures (PDB: 8F8T), only the terminal subunit remains twisted, while P-1 adopts a conformation more similar to filamentous actin^44^. This flattening is even more pronounced in the spectrin-actin complex (PDB: 8IAH), where both P and P-1 are flattened (Supplementary Table 2). Tropomyosin and SH3BGRL-2, in addition to Tmod, may contribute to stabilizing this flat conformation.

While we observed only weak SH3BGRL-mediated actin nucleation *in vitro*, we found that SH3BGRL and SH3BGRL-3 are potent inhibitors of actin filament pointed end elongation. Nanomolar concentrations of both proteins are sufficient to efficiently suppress pointed end elongation to a degree comparable to the well-characterized pointed end capping protein Tmod3. In contrast to Tmod3, however, SH3BGRL and SH3BGRL-3 are far less effective at protecting the pointed end from depolymerization, as micromolar concentrations are required to observe appreciable inhibition of pointed end subunit loss. These observations suggest that binding of SH3BGRL proteins to the pointed end is sufficient to sterically block subunit addition under conditions favouring polymerization, but that their interaction with the terminal actin protomers is insufficient to substantially slow subunit dissociation under depolymerizing conditions. Future studies will be needed to determine the extent to which the interaction of SH3BGRL proteins with the pointed end is influenced by the nucleotide state of the terminal subunits, which differs between assays: during pointed end elongation, the terminal subunits are predominantly ATP- or ADP-P_i_-actin, whereas during pointed end depolymerization, they consist primarily of ADP-actin.

Our *in vitro* experiments with SH3BGRL/-3 and Tmod3 present simultaneously, together with analysis of the existing structural data of SH3BGRL-2 at the pointed end, suggest that both SH3BGRL isoforms can occupy the pointed end in conjunction with tropomodulin, most likely in a structural arrangement similar to that reported for SH3BGRL-2 and Tmod1^14^. Therefore, we suggest that this is a common feature among the SH3BGRL protein family. In our experiments, we observed isoform-specific differences between SH3BGRL and SH3BGRL-3. Although both isoforms more effectively reduced subunit loss when combined with Tmod3 than when acting alone, SH3BGRL additionally displayed an increased apparent affinity for the pointed end in the presence of Tmod3. One possible explanation is the C-terminal extension unique to SH3BGRL, which may form additional contacts with tropomodulin when both proteins are simultaneously bound to the pointed end (Fig. 8E). Further structural and mutational analyses will be needed to define these isoform-specific differences and to elucidate how Tmod and SH3BGRL proteins jointly regulate cellular actin dynamics. Interestingly, a recent study using co-immunoprecipitation followed by mass spectrometry identified SH3BGRL proteins and tropomodulins as components of the membrane-associated periodic skeleton in mouse neurons, suggesting that this protein family may also cooperate with tropomodulins in other spectrin–actin-based protein complexes^45^.

Our experimental observations, together with recent structural studies, define a clear mechanistic role for SH3BGRL proteins as actin-binding proteins. We therefore propose a new name for this protein family: **t**hioredoxin fold-containing **p**ointed **e**nd **c**apping proteins (TPECs). This suggestion is based on the following considerations: **(1)**The name SH3BGRL originates from a singular SH3-binding motif in SH3BGRL and SH3BGRL-2, which is absent in SH3BGRL-3. Structural analyses, including our own, indicate that this motif is buried within the tertiary structure of SH3BGRL and SH3BGRL-2, making direct interactions unlikely^9^. Moreover, no experimental evidence currently supports SH3 domain binding by these proteins. Thus, the existing name may be misleading. **(2)** Our in vitro experiments demonstrate that SH3BGRL proteins efficiently inhibit actin subunit addition at the pointed end. Structural data and modeling support direct binding to the pointed end of actin filaments, qualifying these proteins as pointed end capping proteins. **(3)** The thioredoxin fold is the defining structural feature of the SH3BGRL family and is also present in the muscle-specific SH3BGR, which was not analyzed here. Furthermore, *Saccharomyces cerevisiae* Aip5 appears to bind the pointed end via a similar mechanism, implying that a subset of thioredoxin fold proteins may be evolutionarily conserved actin-binding proteins across kingdoms, as also suggested by Magliozzi et al. in their recent study on Aip5 function^46^.

Following the new naming conventions, SH3BGRL, SH3BGRL-2, and SH3BGRL-3 would be renamed TPEC, TPEC-2, and TPEC-3, respectively.

In conclusion, our study provides key mechanistic insights into the involvement of the SH3BGRL protein family/TPECs in actin dynamics and sheds light on an expanded function of the thioredoxin fold beyond its conventional enzymatic function, demonstrating its potential as a critical regulatory component in cytoskeletal organization.

## Materials and methods

### Plasmids

The coding sequences of human SH3BGRL (Uniprot-ID: O75368), human SH3BGRL-2 (Uniprot-ID: Q9UJC5) and human SH3BGRL-3 (Uniprot-ID: Q9H299) were obtained from the Uniprot database, and the corresponding cDNA sequences were synthesized using the GeneArt Gene Synthesis service (Thermo Fisher Scientific, Waltham, MA, USA). The coding sequences were cloned into the pGEX-6P-2 vector for expression as GST-fusion proteins and verified via overnight sequencing. The coding sequence of human cytoskeletal β-actin (Uniprot-ID: P60709) was fused via a C-terminal linker (ASR(GGS)_3_A) to a His_8_-tagged thymosin-β4 moiety (Uniprot-ID: P62328) and cloned into the multiple cloning site of the pFastBac-Dual vector under the control of the polyhedrin promotor.

### Protein production and purification

Human GST-SH3BGRL/-2/-3 fusion proteins were produced in Rosetta2 cells. For a typical preparation, cells from 1 L of expression culture were resuspended in 100 mL lysis buffer (25 mM HEPES pH 7.4, 150 mM NaCl, 15 mM CaCl_2_, 1 mM DTT, 1 mM PMSF, 100 µg/mL TAME, 80 µg/mL TPCK, 2 µg/mL pepstatin, 5 µg/mL leupeptin) supplemented with 250 µg/mL lysozyme (from hen egg white; Merck KGaA, Darmstadt, Germany) and incubated on ice for 30 min. The cells were lysed by sonification and treated with DNase-I (Roche, Basel, Switzerland) for 30 min on ice to remove bacterial DNA. The lysate was cleared by centrifugation at 35,000 × *g* for 30 min and the cleared supernatant was subsequently loaded onto a self-packed GSH-Sepharose column and washed with 5 column volumes of wash buffer (25 mM HEPES pH 7.4, 150 mM NaCl, 1 mM DTT, 1 mM EDTA). The fusion protein was digested on-column with PreScission protease overnight at 4 °C. On the next day the SH3BGRL/−2/−3 was eluted from the column with wash buffer. The protein was concentrated and loaded onto a S75 16/600 size exclusion chromatography column (GE Healthcare, Chicago, IL, USA) equilibrated with SEC-buffer (25 mM HEPES pH 7.4, 50 mM NaCl, 0.5 mM TCEP). Fractions containing pure protein were pooled, concentrated, frozen in liquid nitrogen and stored at −80 °C until used further. Purification of human cytoskeletal β-actin and labelling at the accessible C-terminal cysteine with ATTO-655 (ATTO-TEC, Siegen, Germany) was performed exactly as previously described^47^. α-skeletal actin (UniProt ID: P68139) was prepared from chicken pectoralis major muscle and labeled at the accessible C-terminal cysteine with *N*-(1-pyrene)iodoacetamide (Thermo Fisher Scientific, Waltham, MA, USA) or ATTO-488 (ATTO-TEC, Siegen, Germany) following previously described protocols^48–50^. To further purify actin for use in polymerization studies and for analytical ultracentrifugation, the crude G-actin solution was centrifuged (186,000 × *g*, 2 h, 4 °C) and loaded onto a S75 16/600 size exclusion chromatography column (GE Healthcare, Chicago, IL, USA) equilibrated with G-buffer (10 mM TRIS pH 8.0, 0.2 mM CaCl_2_, 0.2 mM ATP, 0.5 mM DTT). Only fractions from the second half of the chromatography peak were used for polymerization studies and analytical ultracentrifugation. Production and purification of human thymosin-β4-His_8_^47^, human cofilin-1^51^, human profilin-1^51^, mouse SNAP-mDia1(FH1FH2)-His_6_^52^ and human tropomodulin-3^53^ were performed as previously described.

### Nucleotide exchange assay

The rate constant of ATP dissociation (*k*_− T_) for monomeric cytoskeletal β-actin in the absence and presence of SH3BGRL-2, profilin-1, thymosin-β4-His_8_ and cofilin-1 was determined using ε-ATP (Jena Bioscience, Jena, Germany) at a HiTech Scientific SF61 stopped-flow system (TgK Scientific Limited, Bradford on Avon, UK), as previously described^51^. For experiments in the presence of actin-binding proteins (ABPs), actin and the respective ABP were incubated on ice for 15 min prior to experiment.

### Analytical size-exclusion chromatography

Freshly purified monomeric skeletal α-actin was treated with a 1.5 molar excess of latrunculin B (LatB) for use in analytical size-exclusion chromatography. LatB-actin and SH3BGRL-3 were diluted into G-buffer to the indicated concentrations and a final volume of 100 µL and incubated on ice for 15 min. The mixture was injected onto a S75 10/300 size-exclusion chromatography column (GE Healthcare, Chicago, IL, USA) equilibrated with G-buffer (10 mM TRIS pH 8.0, 0.2 mM CaCl_2_, 0.5 mM DTT).

### Pyrene-actin based bulk-polymerization assays

Pyrene-actin based bulk-polymerization experiments in the absence and presence of SH3BGRL proteins were performed at a CLARIOstar Plus microplate reader (BMG LABTECH, Offenburg, Germany) at 25 °C. Pyrene-labeled Mg^2+^-ATP-G-actin (5% labeled) was premixed with SH3BGRL proteins. 20 µL of the individual reaction mixtures were placed in a black flat- bottom 96-well plate (BrandTech Scientific, USA). 80 µL of 1.25× polymerization buffer (10 mM HEPES pH 7.4, 50 mM KCl, 5 mM MgCl_2_, 0.5 mM EGTA, 0.1 mM DTT, and 0.1 mM ATP) was added to the wells using the built-in pipetting function of the plate reader, and the polymerization of actin was followed as an increase in pyrenyl-fluorescence. For experiments in the presence of profilin-1 and mDia1, all components were premixed on ice and then placed in the 96-well plate. The bulk-polymerization rates were determined by linear regression analysis of the linear region around the time-point of half-maximal fluorescence^54^. The lag-time (*t*_lag_) of the reaction was defined as the time-point at which the reaction reaches 5% of the final fluorescence signal.

To determine the concentration of filament ends over the time course of the experiment, we used the following approach. The association rate constant *k*_on_ obtained from our TIRFM experiments in the absence of SH3BGRL proteins was used to calculate the filament end concentration under all conditions, as we did not observe any significant change in *k*_on_ in the presence of SH3BGRL proteins. For the dissociation rate constant, we used the published value (*k*_off_ = 1.4 s^− 1^)^55^. The total actin concentration in the assay was 2 µM. The concentration of filament ends was then calculated using the following equation:

$$\:\left[\mathrm{f}\mathrm{i}\mathrm{l}\mathrm{a}\mathrm{m}\mathrm{e}\mathrm{n}\mathrm{t}\:\mathrm{e}\mathrm{n}\mathrm{d}\mathrm{s}\right]=\frac{\frac{\mathrm{d}{\mathrm{F}}_{\mathrm{n}\mathrm{o}\mathrm{r}\mathrm{m}}}{\mathrm{d}\mathrm{t}}\cdot\:\left({\left[\mathrm{a}\mathrm{c}\mathrm{t}\mathrm{i}\mathrm{n}\right]}_{\mathrm{t}\mathrm{o}\mathrm{t}\mathrm{a}\mathrm{l}}-\frac{{k}_{\mathrm{o}\mathrm{f}\mathrm{f}}}{{k}_{\mathrm{o}\mathrm{n}}}\right)}{{\mathrm{k}}_{\mathrm{o}\mathrm{n}}\cdot\:\left({\left[\mathrm{a}\mathrm{c}\mathrm{t}\mathrm{i}\mathrm{n}\right]}_{\mathrm{t}\mathrm{o}\mathrm{t}\mathrm{a}\mathrm{l}}\cdot\:\left(1-{\mathrm{F}}_{\mathrm{n}\mathrm{o}\mathrm{r}\mathrm{m}}\right)+\frac{{k}_{\mathrm{o}\mathrm{f}\mathrm{f}}}{{k}_{\mathrm{o}\mathrm{n}}}\cdot\:{\mathrm{F}}_{\mathrm{n}\mathrm{o}\mathrm{r}\mathrm{m}}\right)-{k}_{\mathrm{o}\mathrm{f}\mathrm{f}}}$$

where F_norm_ is the normalized pyrenyl-fluorescence (ranging from 0 to 1), [actin]_total_ is the total actin concentration in the assay (2 µM), and *k*_on_ and *k*_off_ are the association (10.3 µM^− 1^ s^− 1^) and dissociation rate constants (1.4 s^− 1^), respectively. Plotting the calculated filament end concentration over the time course of the experiment yields the curves shown in Fig. 5B.

Pointed end elongation assays were performed under conditions similar to the bulk-polymerization experiments, with modifications based on the procedure described in^56^. Briefly, 0.8 µM α-actin (F-actin) mixed with 8 nM capping protein served as actin seeds and were incubated with varying concentrations of SH3BGRL, SH3BGRL-3, and Tmod3. Polymerization was initiated by adding 2 µM G-actin (8% labeled) together with 157 µl of 1.27× KMEI buffer (final concentrations: 10 mM HEPES pH 7.4, 50 mM KCl, 1 mM MgCl_2_, 1 mM EGTA). The initial linear phase of the fluorescence traces was fitted to determine the elongation rate.

For depolymerization experiments specifically probing the pointed end, pyrene-labeled actin (10 µM, 20% labeled) was polymerized overnight in the presence of 0.1 µM human capping protein (CP) to generate CP-capped actin filaments. 10 µM of pre-polymerized pyrene-labeled CP-capped F-actin (20% labeled) was incubated with SH3BGRL proteins on ice for 15 min. The reaction mixtures were rapidly diluted to 0.5 µM and the depolymerization of the filaments from the pointed ends was tracked via the decrease in pyrenyl-fluorescence. Mono-exponential functions were used to determine the apparent rate constant of pointed end depolymerization under the various conditions. The normalized depolymerization rate constants were plotted against the protein concentration used to estimate the respective *K*_D_/*K*_50%_ values via a hyperbolic function:

$$\:\mathrm{y}={\mathrm{y}}_{0}+({\mathrm{y}}_{\mathrm{m}\mathrm{a}\mathrm{x}}-{\mathrm{y}}_{0})\cdot\:\frac{{\mathrm{x}}^{\mathrm{n}}}{{K}_{\mathrm{D}}^{\mathrm{n}}+{\mathrm{x}}^{\mathrm{n}}}$$

with $$\:{\mathrm{y}}_{0}=1,\:\mathrm{n}=1$$.

### TIRF microscopy-based assays

The effect of SH3BGRL proteins on filament nucleation and elongation was assessed using TIRF microscopy-based assays with freshly clarified ATTO-488 labeled α-actin and ATTO-655 labeled cytoskeletal β-actin. Glass coverslips used for flow-cell assembly were cleaned and chemically treated according to previously described protocols^51^. Actin polymerization was initiated by diluting the G-actin solution (containing 15% labeled actin (α-actin) or 10% labeled actin (β-actin)) to a final concentration of 1 µM in 2×TIRF-buffer (20 mM HEPES pH 7.4, 50 mM KCl, 1 mM MgCl_2_, 1 mM EGTA, 0.2 mM ATP, 15 mM glucose, 20 mM β-ME, 0.25% methylcellulose, 0.1 mg/mL glucose oxidase and 0.02 mg/mL catalase). After mixing, the solutions were immediately flushed into flow-cells and image acquisition was started. For experiments involving SH3BGRL isoforms, the isoforms were pre-diluted in KMEI buffer (10 mM imidazole pH 7.4, 50 mM KCl, 1 mM MgCl_2_, 1 mM EGTA) and further diluted to the final concentration in TIRF-buffer before the addition of actin.

Image series were acquired at an Olympus IX83 inverted fluorescence microscopes (Olympus, Hamburg, Germany) equipped with a 60×/1.49 NA PlanApo TIRF oil immersion objective and an Orca Flash 4.0 CMOS camera (Hamamatsu Photonics Deutschland GmbH, Herrsching, Germany). Nucleation of actin filaments was analyzed by automated image analysis using the *Analyze particles* plugin in ImageJ^57^. The elongation rates of individual actin filaments were measured by manually tracking their growth.

### AlphaFold

AlphaFold3^32^ and AlphaFold-Multimer (v3) were used to predict potential heterodimeric complexes of human SH3BGRL proteins and human cytoskeletal β-actin. The respective primary sequences were retrieved from the Uniprot database (see “Plasmids”). Predictions using AlphaFold3 were performed using the AlphaFold server (https://golgi.sandbox.google.com). We ran 10 jobs on random-seed for each complex combination resulting in 50 models in total for each combination. The metrics for further evaluation of the complexes were extracted from the corresponding JSON files. Predictions using AlphaFold-Multimer (v3) with and without templates were performed on the Halime HPC cluster at the Institute for Biophysical Chemistry (Hannover Medical School, Germany). We generated a total of 25 complex predictions for each complex combination. Metrics for further analysis were extracted from the AlphaFold pickle files of each respective prediction via custom python scripts.

Visualization of the predicted complex structures was performed using ChimeraX^13^.

### Molecular dynamics (MD) simulations

The individual complexes of the F-actin pointed end with the different SH3BGRL isoforms were modelled by positioning the AlphaFold3 generated^32^ models of the SH3BGRL isoforms in the binding orientation observed for SH3BGRL-2 in the experimental structure of the spectrin–actin complex (PDB: 8IAH). Since SH3BGRL isoforms interact exclusively with two actin subunits, each simulation system was simplified to contain one SH3BGRL isoform bound to the two interacting actin subunits. Molecular dynamics simulations were performed using the GROMACS 2024.3^58^ simulation package with the CHARMM36m^59^ force field. Each protein complex was placed in a triclinic box with a minimum distance of 1.2 nm between the protein and the box edges to prevent periodic image interactions. The systems were solvated with TIP3P water molecules, and sodium and chloride ions were added to a final concentration of 0.15 M. Additional sodium ions were introduced to neutralize the overall charge of each system. Energy minimization was performed using the steepest descent algorithm to remove steric clashes and unfavourable interactions. The equilibration process was performed in two phases: first, a 100 ps NVT (constant number of particles, volume, and temperature) equilibration was performed to stabilize the temperature at 300 K, followed by a 100 ps NPT (constant number of particles, pressure, and temperature) equilibration using the Parrinello-Rahman barostat to maintain the pressure at 1 bar. For the production simulations, the backbone atoms of both actin subunits were position-restrained with a force constant of 5000 kJ/mol·nm² in all dimensions to maintain the structural integrity of the F-actin pointed end and prevent reorientation during the simulation. Production runs were performed for 500 ns at 300 K using the leap-frog integrator with a 2 fs time step. All bonds involving hydrogen atoms were constrained using the LINCS algorithm. Long-range electrostatic interactions were calculated using the Particle Mesh Ewald method with a real-space cut-off of 1.2 nm, while van der Waals interactions were truncated at 1.2 nm with a force-switch smoothing function applied from 1.0 nm. Coordinates were saved every 10 ps for subsequent analysis.

The root mean square deviation (RMSD) and root mean square fluctuation (RMSF) of the simulation were calculated using the corresponding GROMACS function. The interface analysis was performed using the Python libraries MDAnalysis^60^ and ProLIF^61^. The contacts obtained by ProLIF were filtered and transformed into the specific json structure required as Flareplot input using a custom Python script. Visualization of the molecular structure was performed using ChimeraX 1.9^13^.

### Analytical ultracentrifugation

Sedimentation velocity runs were carried out in an Optima AUC analytical ultracentrifuge (Beckman Coulter, USA) using an An-50 Ti rotor at 50,000 rpm and 20 °C. Concentration profiles were measured with the absorption scanning optics at 280 nm–230 nm using 3–12 mm standard double-sector centerpieces filled with 100 µL or 400 µL sample, respectively. Experiments to determine the oligomerization state of SH3BGRL, SH3BGRL-2 and SH3BGRL-3 were performed in a buffer containing 25 mM HEPES pH 7.4, 50 mM NaCl, 0.5 mM TCEP in the indicated concentration range. To test whether SH3BGRL or SH3BGRL-3 are able to interact with G-actin, 10 µM actin supplemented with 15 µM latrunculin B was titrated with the indicated amounts of these proteins in a buffer containing 9 mM Tris pH 7.8, 50 mM KCl, 5 mM MgCl_2_, 0.18 mM CaCl_2_, 45 µM ATP, and 0.18 mM TCEP. After mixing, all samples were allowed to equilibrate for 4 h at 20 °C prior to sedimentation analysis.

For data analysis a model for diffusion-deconvoluted differential sedimentation coefficient distributions (continuous c(s) distributions) implemented in the program SEDFIT^30^ was used. Buffer density, viscosity and partial specific volumes were calculated from the buffer and amino acid composition, respectively, using the program SEDNTERP^62^ and were used to correct the experimental s-values to s_20,w_. In the case of mixtures of G-actin and SH3BGRL/-3, experimental s-values are given as s_20,w_ correction is not appropriate due to the different partial specific volumes of the proteins. Figures showing c(s) distributions were obtained using the program GUSSI^63^.

Protein concentrations were determined spectrophotometrically using the absorption coefficients at 280 nm as calculated from amino acid composition^64^ and are given in monomers throughout the text.

### Data analysis

Data analysis and graph plotting were performed with Origin 2024 (OriginLab Corporation. Massachusetts, USA). Errors are given as standard deviation (SD) based on three independent experiments if not otherwise specified. The significance of the data was evaluated in Origin 2024 using a two-sample t-test (*p* > 0.05 ≙ ns, *p* ≤ 0.05 ≙ *, *p* ≤ 0.01 ≙ **, *p* ≤ 0.001 ≙ ***, *p* ≤ 0.0001 ≙ ****).

## Supplementary Information

Below is the link to the electronic supplementary material.

Supplementary Material 1

## Data Availability

All data generated or analysed during this study are included in this published article and its supplementary information files. The raw datasets are available from the corresponding author on reasonable request.

## Materials availability

Unique reagents generated in this study are available from the lead contact with a completed Materials Transfer Agreement.
