# Supplementary material for: SH3BGRL proteins are thioredoxin fold–containing actin filament pointed end capping proteins (TPECs)
Source: Sci Rep. 2026 Jan 21;16:3038. doi: 10.1038/s41598-025-34096-y (PMC12827327; doi:10.1038/s41598-025-34096-y)
Supplement: Supplementary file 1 — Supplementary Material 1 [file 41598_2025_34096_MOESM1_ESM.docx]

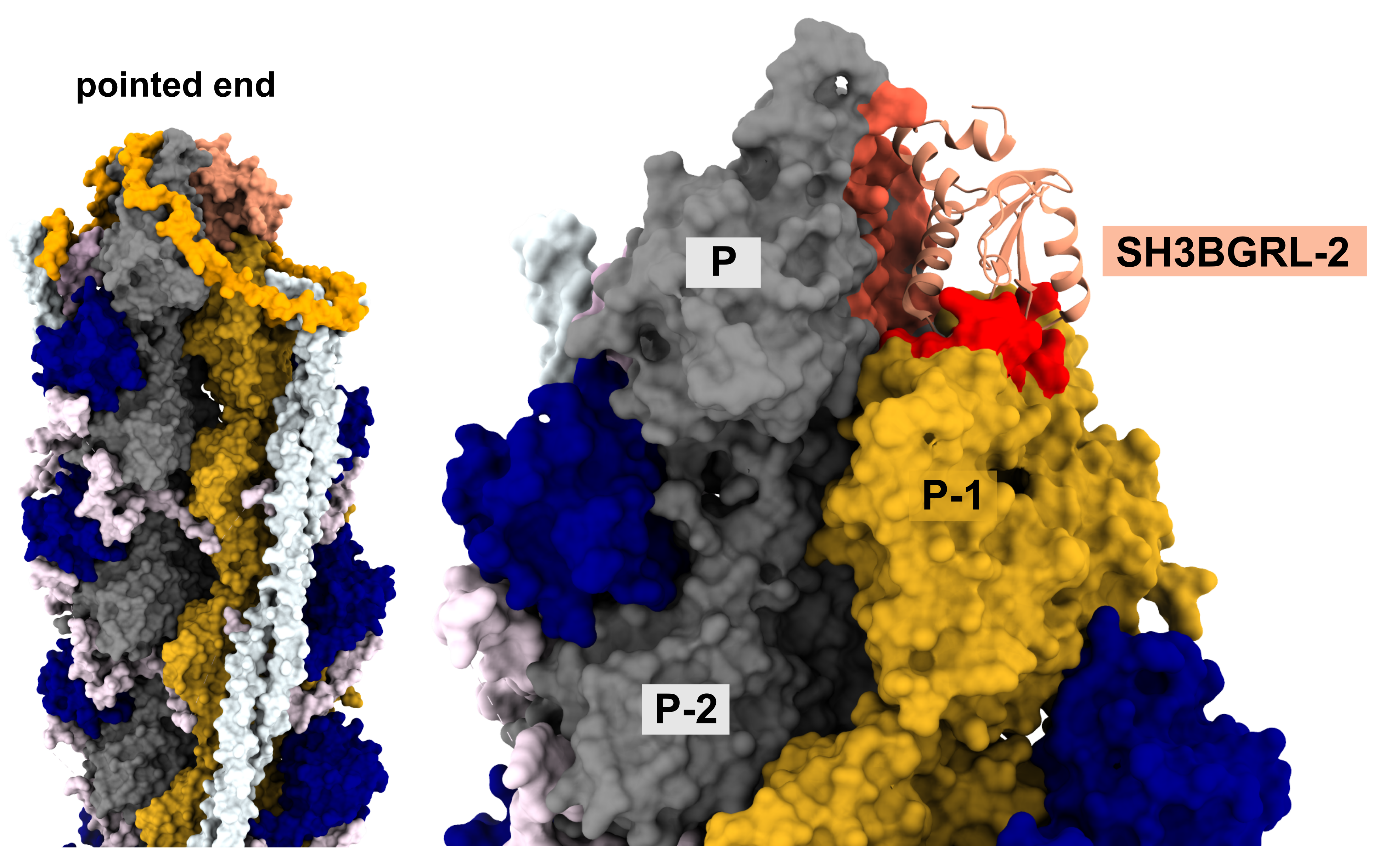


**Supplementary Figure 1: Structural overview of the spectrin–actin complex purified from porcine erythrocytes (PDB: 8IAH).**

**(Left)** Model of the pointed end of the spectrin–actin complex comprising spectrin (navy), actin (grey and gold), SH3BGRL2 (pink), tropomyosin (light blue), dematin (rose), and tropomodulin-1 (orange). **(Right)** Close-up view of SH3BGRL-2 binding at the actin pointed end; tropomodulin and tropomyosin were omitted for clarity. The buried surface area between the terminal (P) and penultimate (P-1) actin protomers is highlighted in red.


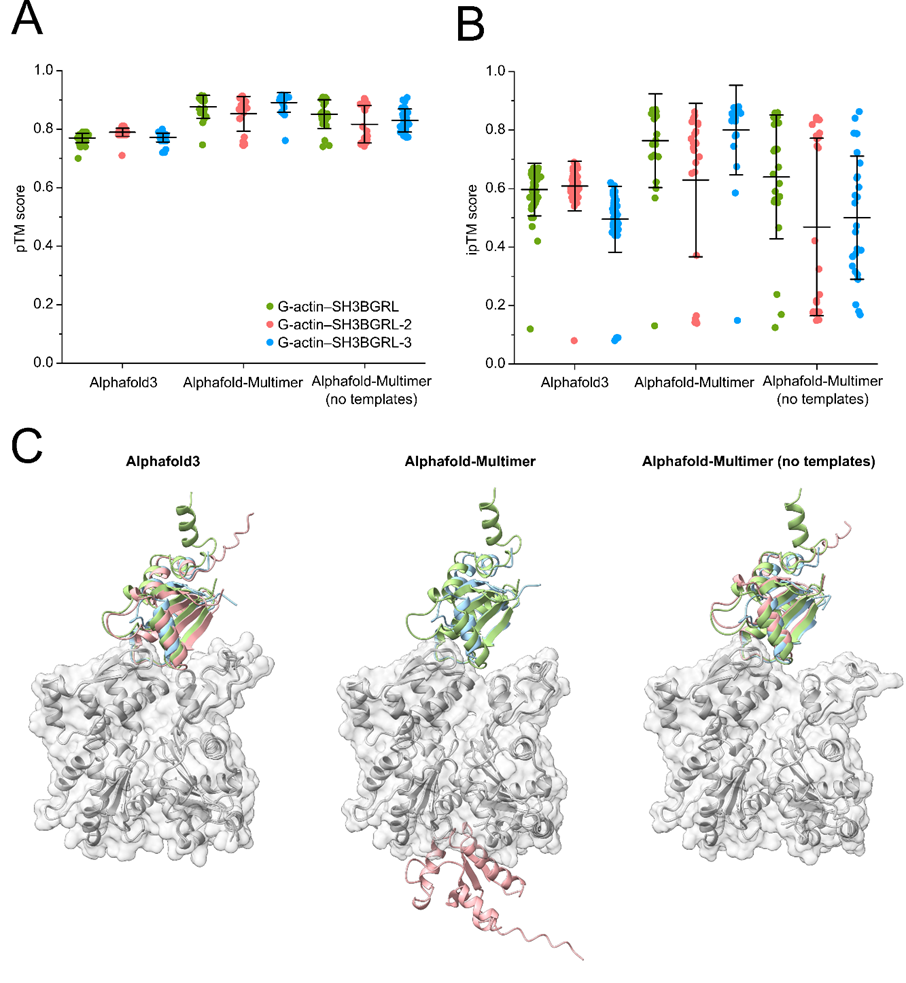


**Supplementary Figure 2: Investigation of potential SH3BGRL–G-actin complexes using AlphaFold**

**(A)** Plotted predicted Template Modeling (pTM) scores for predictions of heterodimeric complexes of human SH3BGRL proteins with monomeric human β-actin derived using the indicated AlphaFold version. **(B)** Plotted interface predicted Template Modeling (ipTM) scores of the predictions shown in (A). **(C)** Overlay of the best ranked models of the SH3BGRL–G-actin complexes (SH3BGRL in green, SH3BGRL-2 in red, SH3BGRL-3 in blue, β-actin in grey) derived from the different used AlphaFold versions

We initially evaluated models generated by AlphaFold3 by plotting their respective predicted template modelling (pTM) and the interface predicted template modelling (ipTM) scores, which are metrics evaluating the accuracy of the predicted folding and the interaction interface, respectively. (Figure 3A, B). The pTM scores for all predictions ranged between 0.7 and 0.8, indicating a reliable prediction of the individual folds of the complex partners. In contrast, the ipTM scores revealed either failed or low-confidence predictions for the interactions within the complex. The most prominent complex predictions place the SH3BGRL proteins at the pointed end of the actin monomer (Figure 3C), closely resembling the arrangement of the penultimate actin protomer and SH3BGRL-2 in the available cryoEM-structure of the SH3BGRL-2 decorated actin filament [20] (Suppl. Figure 1). This indicates bias of AlphaFold3 towards this particular structural arrangement, likely due to inclusion of this structure in the training dataset or to the use of structural templates. To reduce this potential bias, we repeated the predictions using AlphaFold-Multimer, both with and without the use of templates (Figure 3A, B). These new predictions yielded a wider range of complex conformations. Under both conditions, AlphaFold-Multimer produced high-confidence predictions (ipTM > 0.8). However, most of these high-confidence predictions again placed SH3BGRL proteins at the pointed end of the actin monomer, similar to the conformations predicted by AlphaFold3. An exception was SH3BGRL-2, which was confidently positioned at the barbed end of the actin monomer in predictions using AlphaFold-Multimer with structural templates (Figure 3C).

**
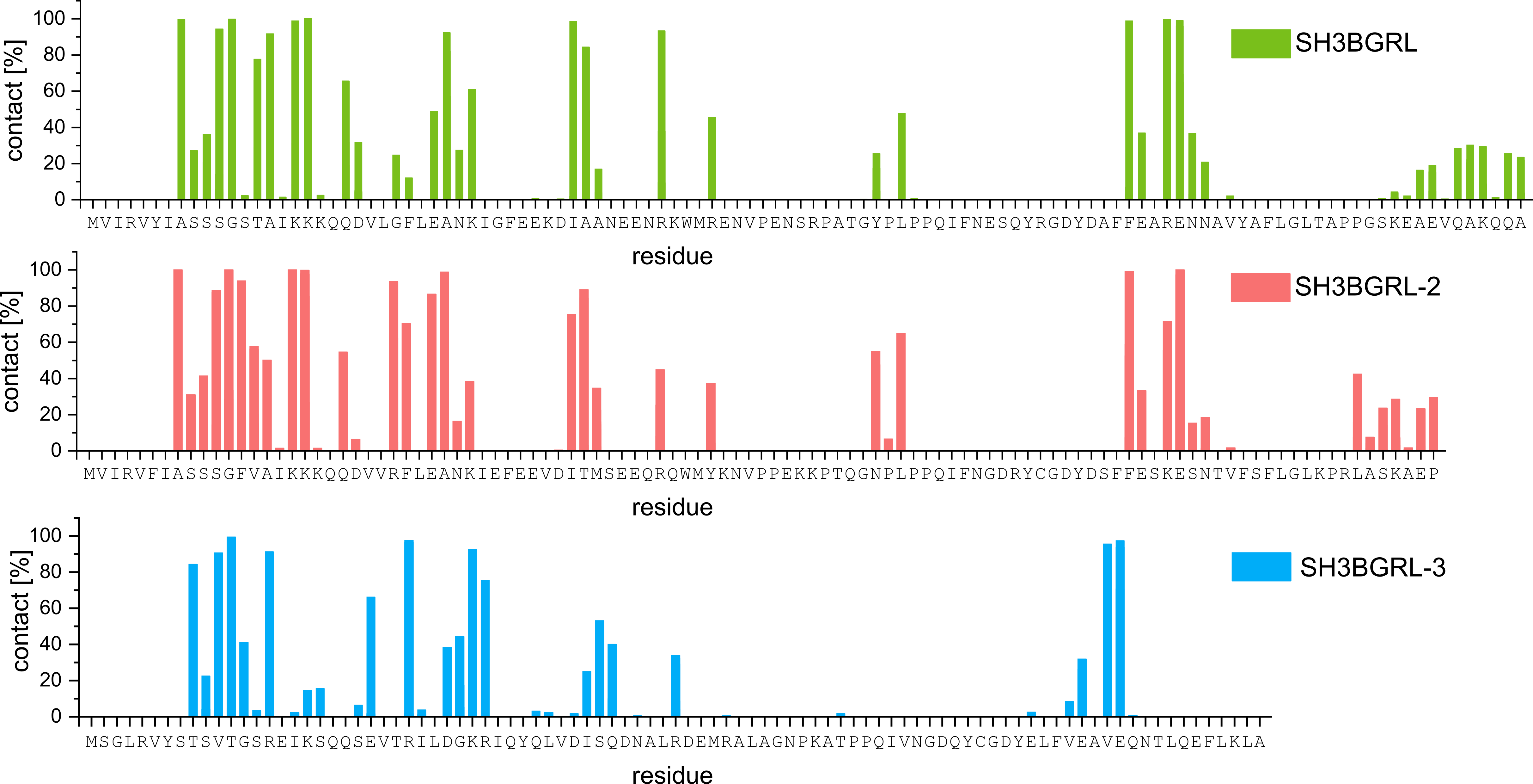
**

**Supplementary Figure 3: Contact frequency during molecular dynamics simulations**

Bar plots of contact frequencies, representing the fraction of the simulation time that the specific residue was in contact with actin.

**
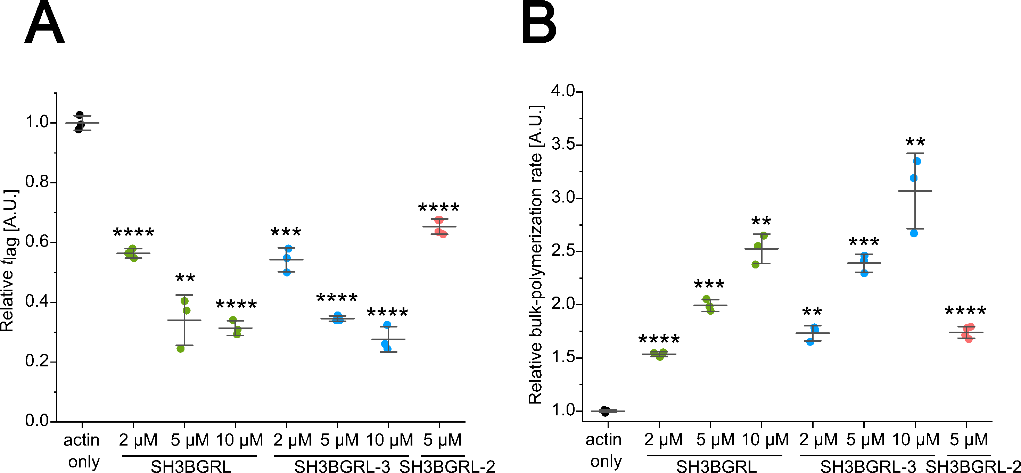
**

**Supplementary Figure 4: Determination of *t*_lag_ and the bulk-polymerization rate from pyrene-actin based polymerization assays**

**(A)** Relative values of *t*_lag_ (to actin only experiments) determined from experiments shown in Figure 5A. *t*_lag_ was determined as the time-point at which the reaction reaches 5% of the final fluorescence signal. Data are shown as the mean ± SD of all performed experiments. N=3 for each condition. Significance is given compared to actin only experiments (p > 0.05 ≙ ns, p ≤ 0.05 ≙ *, p ≤ 0.01 ≙ **, p ≤ 0.001 ≙ ***, p ≤ 0.0001 ≙ ****). **(B)** Relative bulk-polymerization rates (to actin only experiments) determined from the experiments shown in Figure 5A. Data are shown as the mean ± SD of all performed experiments. N=3 for each condition. Significance is given compared to actin only experiments (p > 0.05 ≙ ns, p ≤ 0.05 ≙ *, p ≤ 0.01 ≙ **, p ≤ 0.001 ≙ ***, p ≤ 0.0001 ≙ ****).

**
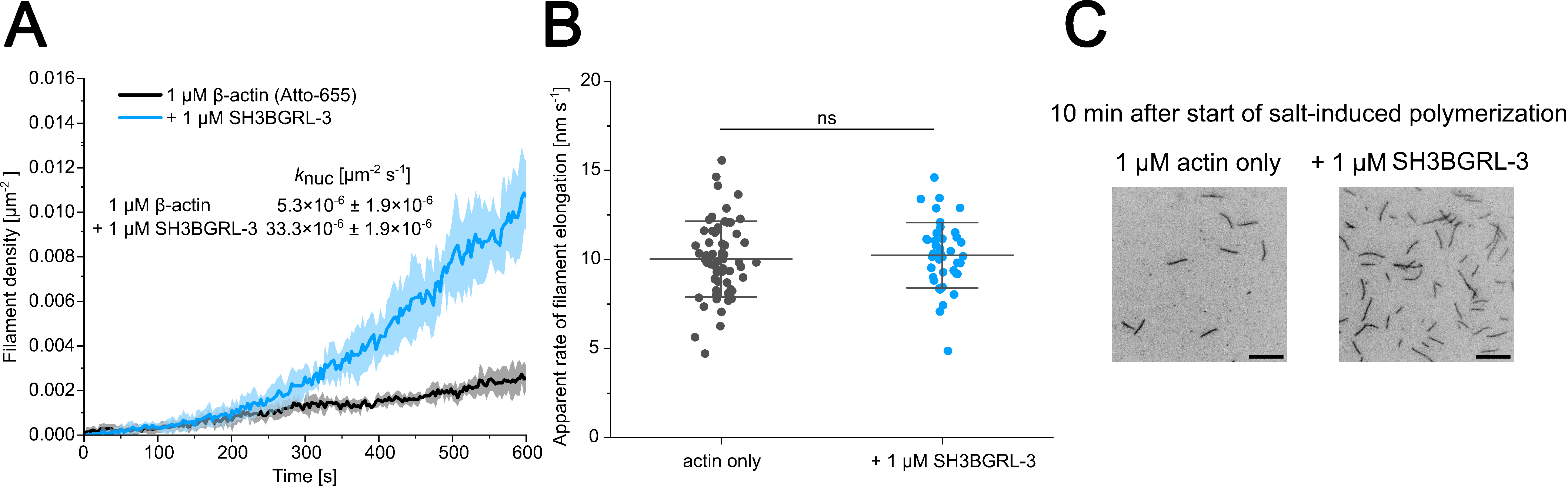
**

**Supplementary Figure 5: Analysis of the effect of SH3BGRL-3 on nucleation and elongation of recombinant human β-actin using *in vitro* TIRF microscopy**

**(A, B)** Polymerization of 1 µM fluorescently labeled β-actin (10% Atto-655 labeled) was induced by salt-shift in the absence or presence of 1 µM SH3BGRL-3. The progression of the reaction was tracked by TIRF microscopy. Nucleation efficiency and filament elongation were measured as described in Figure 8. The solid lines and shades in A represent the mean ± SD of at least three individual experiments. Every data point in B represents an individual filament. Data is shown as the mean ± SD. **(C)** Representative micrographs of TIRFM-based polymerization experiments under the indicated conditions 10 min after induction of polymerization. Scale bar corresponds to 10 µm.

**
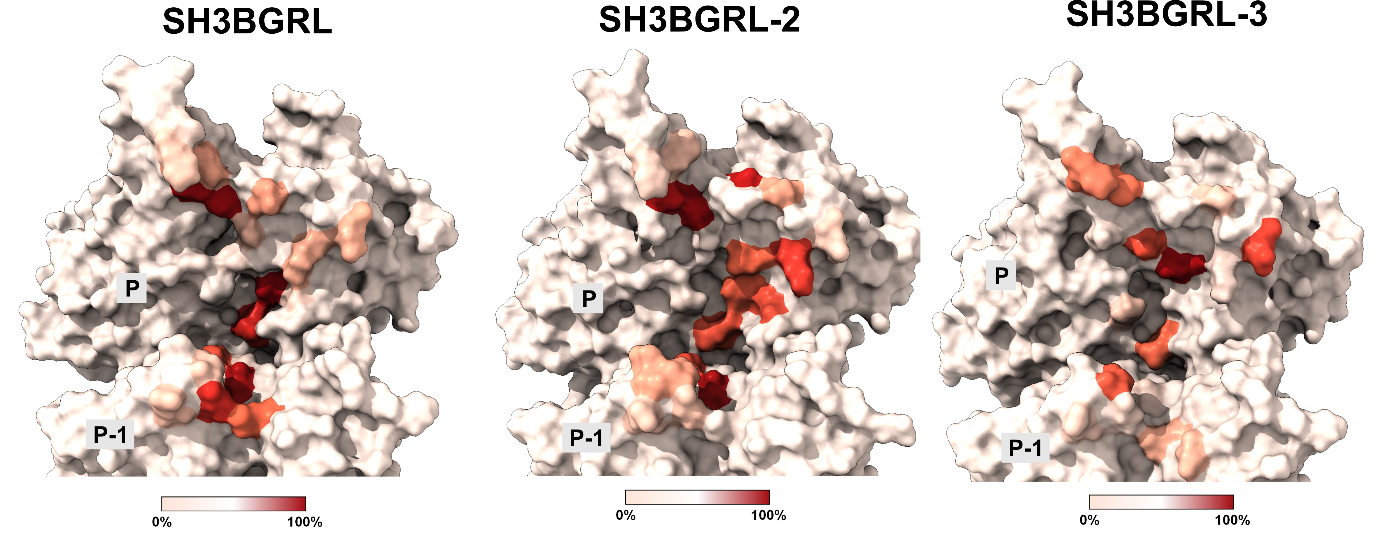
**

**Supplementary Figure 6: Analysis of the contact interfaces of SH3BGRL, SH3BGRL-2, and SH3BGRL-3 with the penultimate (P–1) and terminal (P) actin subunits.**

Both actin subunits are shown and colored according to their contact frequency with the respective SH3BGRL isoform, as indicated above. Contact frequencies were calculated over the final 150 ns of the MD simulation and are represented by a red color scale, where increasing intensity denotes higher contact frequency (see color bar below).

**Supplementary Table 1** Interactors of human SH3BGRL/-2/-3 deposited in the BioGRID database. Only interactors canonically associated with the actin cytoskeleton are shown. Interactors are indicated by their gene name. Database access date: December 2024.

| **SH3BGRL** | **SH3BGRL-2** | **SH3BGRL-3** |
| --- | --- | --- |
| \| ACTB \| \| --- \| \| ACTBL2 \| \| ACTG1 \| \| ARPC1A \| \| ARPC1B \| \| ARPC2 \| \| ARPC3 \| \| ARPC4 \| \| ARPC5 \| \| ARPC5L \| \| CAPZA1 \| \| CAPZA2 \| \| CAPZB \| \| CFL1 \| \| CORO1B \| \| CORO1C \| \| CORO2A \| \| COTL1 \| \| CTTN \| \| DBN1 \| \| DSTN \| \| GSN \| \| LIMA1 \| \| MYH14 \| \| MYO1B \| \| MYO1C \| \| MYO1D \| \| MYO1E \| \| MYO5A \| \| MYO5B \| \| MYO5C \| \| MYO6 \| \| PFN1 \| \| PLS3 \| \| TMOD3 \| \| TWF1 \| \| TWF2 \| \| WDR1 \| | \| ACTB \| \| --- \| \| CAPZB \| \| GSN \| \| MYO1D \| \| MYO5A \| \| MYO5C \| \| TMOD1 \| \| TMOD2 \| \| TMOD3 \| | **-** |

**Supplementary Table 2** Dihedral angles between subdomain 1,2 and subdomain 3,4 of various actin structures.

|  | **PDB code** | **Description** | **P[°]** | **P-1[°]** | **P-2[°]** | **P-3[°]** |
| --- | --- | --- | --- | --- | --- | --- |
| G-actin | 3TU5 | Monomeric actin (for comparison to Zsolnay et al. PNAS 2020) | -14.92 |  |  |  |
| F-actin | 6DJM | F-actin (for comparison to Zsolnay et al. PNAS 2020) | 4.74 | 4.74 | 4.74 | 4.74 |
|  | 6ANU | F-actin with spectrin (Avery et al. Nat. Comm. 2017) | 2.52 | 2.52 | 2.52 | 2.52 |
| Pointed end | 8IAH | Spectrin-actin complex with SH3BGRL-2 | -0.45 | -0.42 | 3.90 | 4.70 |
|  | 8F8S | Free Pointed end (Carman et al. Science 2023) | -16.54 | -17.87 | 5.68 | 5.48 |
|  | 8F8T | Tmod1-bound pointed end (Carman et al. Science 2023) | -17.62 | -3.42 | 6.17 | 5.83 |
|  | 9FJO | Free Pointed end Sanders et al (Nat. Comm. 2024) | -16.90 | -16.75 | 5.26 | 5.28 |
|  | 9FJM | Phalloidin-bound pointed end (Sanders et al. Nat. Comm. 2024) | 3.41 | 3.16 | 5.09 | 4.53 |
